# CAR-T cells and CAR-Tregs targeting conventional type-1 dendritic cell suppress experimental autoimmune encephalomyelitis

**DOI:** 10.3389/fimmu.2023.1235222

**Published:** 2023-10-27

**Authors:** Cody D. Moorman, Sherman Yu, Carlos G. Briseno, Hyewon Phee, Anupama Sahoo, Ambika Ramrakhiani, Ashutosh Chaudhry

**Affiliations:** Amgen Research, Amgen Inc., South San Francisco, CA, United States

**Keywords:** autoimmune disease, autoimmunity, chimeric antigen receptor, CAR-T cells, CAR-Treg, type 1 conventional dendritic cells, XCR1

## Abstract

Conventional type 1 dendritic cells (DC1) contribute to the development of pathogenic T helper type 1 (Th1) cells in part *via* the production of the proinflammatory cytokine interleukin-12. Thus, depletion of DC1 has the potential to dampen autoimmune responses. Here, we developed X-C motif chemokine receptor 1 (XCR1)-specific chimeric antigen receptor (CAR)-T cells and CAR-Tregs that specifically targeted DC1. XCR1 CAR-T cells were successfully generated as CD4^+^ and CD8^+^ T cells, expressed XCR1 CAR efficiently, and induced XCR1-dependent activation, cytokine production and proliferation. XCR1 CAR-T cells selectively depleted DC1 when transferred into RAG2^−/−^ mice with a compensatory increase in conventional type 2 DC (DC2) and plasmacytoid DC (pDC). XCR1 CAR-T cell-mediated depletion of DC1 modestly suppressed the onset of Th1-driven experimental autoimmune encephalomyelitis (EAE), an animal model of multiple sclerosis. Diphtheria toxin-mediated DC1 depletion in XCR1-diphtheria toxin receptor mice also suppressed EAE, suggesting that DC1 depletion was responsible for EAE suppression. XCR1 CAR-Tregs were successfully generated and suppressed effector T cells in the presence of XCR1^+^ cells. Therapeutic treatment with XCR1 CAR-Tregs suppressed Th1-driven EAE. Therefore, we conclude that depletion of DC1 with XCR1 CAR-T cells or immune suppression with XCR1 CAR-Tregs can modestly suppress Th1-driven EAE.

## 1 Introduction

Autoimmunity encompasses over 100 unique diseases, in which an individual’s immune system attacks and destroys self-tissue. An estimated 3–5% of the total population suffers from these collective diseases, with women being affected more than men (1). Multiple sclerosis (MS) is an autoimmune disease of the central nervous system (CNS) and is the leading cause of non-traumatic disability in young adults (2). Evidence suggests that MS is mediated by pathogenic CNS-reactive CD4^+^ T cells and functional deficiencies within the immunosuppressive CD4^+^ regulatory T cell (Tregs) compartment (3). Therefore, therapies that suppress these pathogenic CD4^+^ T cell responses and restore Treg function could in theory constitute a cure for MS. Here, we explore the use of a novel dendritic cell (DC)-targeted chimeric antigen receptor (CAR)-T cell as well as CAR-Treg approach for suppressing pathogenic CD4^+^ T cell immunity and reinforcing the Treg compartment in experimental autoimmune encephalomyelitis (EAE), an animal model of MS.

DCs are a specialized subset of antigen-presenting cells (APC) that orchestrate both immunity and tolerance (4–6). For example, DC ablation can exacerbate autoimmunity (7–9), prevent tumor immunity (10), and suppress viral and bacterial immunity in mice (11, 12). Thus, depending on the context, including the antigen pool (e.g., self or non-self) and environmental cues, DCs orchestrate both pro-inflammatory and anti-inflammatory immune responses. There are three major subsets of DC, namely, conventional type 1 DC (DC1), conventional type 2 DC (DC2), and plasmacytoid DC (pDC). Each of these DC subsets has overlapping yet unique roles that augment the overall immune response. In general, DC1 crosspresent exogenous antigens on MHCI, are required for effective cytotoxic CD8^+^ T-cell responses and support T helper type 1 cell (Th1) immune response *via* the production of interleukin-12 (IL-12). In contrast, DC2 support T helper type 2 cell (Th2) responses, whereas pDC support antiviral immunity *via* the production type I interferon (IFN) (12, 13).

DCs and FOXP3^+^ Tregs are both required for self-tolerance as constitutive depletion of either Tregs (14, 15) or DCs (7) led to the development of spontaneous and fatal autoimmunity. Likewise, transient depletion of Tregs (16) or DCs (8, 9, 17) exacerbated active EAE induced with CFA and neuroantigen. DCs are essential for maintaining Treg homeostasis because increasing DCs expanded Tregs (18–20) whereas DC ablation (17, 18) reduced Treg numbers. In addition, DCs maintain Treg function as Tregs from DC-depleted mice had reduced suppressive capabilities (21).

Tregs in turn act on DCs and directly suppress DC function by reducing the expression of co-stimulatory molecules, antigen-presenting molecules, and inflammatory cytokine secretion, while concurrently increasing the expression of co-inhibitory molecules and immunosuppressive cytokines resulting in the induction of *de novo* Tregs and suppression of T conventional responses (22). For example, Treg-treated DCs have been shown to suppress collagen-induced arthritis (CIA) (23), graft versus host disease (GvHD) (24), and systemic lupus erythematosus (SLE) (24) in mice.

DC depletion has been previously shown to suppress passive EAE induced by encephalitogenic T cell transfer (25–28), but it is unclear whether a particular DC subset plays a prominent role in this setting. While DCs are required for immune tolerance, DC1 might constitute a pathogenic DC subset in certain autoimmune diseases. For example, mice deficient in DC1 were resistant to EAE, type 1 diabetes (T1D), CIA, and postischemic inflammatory heart damage (29–32). In addition, CNS-specific myelin oligodendrocyte glycoprotein (MOG)-loaded DC1 exacerbated EAE in comparison with MOG-loaded DC2 or pDC that ameliorates EAE (33, 34). Therefore, we hypothesized that CAR-T cell-mediated depletion and CAR-Treg-mediated suppression of proinflammatory DC1 would suppress Th1-driven EAE. Likewise, we hypothesized that shifting endogenous antigen presentation to DC2 and pDC might favor Treg induction and immunological tolerance.

To test this idea, we designed a DC1-specific CAR receptor. DC1 were targeted *via* a scFv CAR receptor against the DC1-specific surface protein, X-C motif chemokine receptor (XCR1). The XCR1 CAR was expressed in CD4^+^ and CD8^+^ T cells as well as CD4^+^ Tregs. The XCR1 CAR was functional and engendered T cells with cytotoxic capabilities that led to systemic depletion of XCR1^+^ DC1 *in vivo* without any sign of overt autoimmunity due to the self-reactive CAR receptor. CAR-T cell-mediated depletion of DC1 delayed the onset of passive EAE. Suppression was not due to suppressive functions of CD8^+^ T cells (35) or due to effector T cell competition between encephalitogenic T cells and CAR-T cells since diphtheria toxin (DT)-mediated depletion of DC1 in XCR1-DTR mice also suppressed the onset of EAE to a similar extent. In addition, XCR1 CAR-Tregs responded to XCR1^+^ cells and suppressed passive EAE when administered therapeutically. Overall, we provide proof of concept that CAR-T cells can selectively, systemically, and durably deplete DC1 to modulate autoimmunity and that DC1-targeted CAR-Tregs can suppress autoimmunity.

## 2 Materials and methods

### 2.1 Mice

Female C57BL/6J “CD45.2” (Stock# 000664), B6.SJL-*Ptprc^a^ Pepc^b^*/BoyJ “CD45.1” (Stock# 002014), B6.Cg-Foxp3tm2Tch/J “FIG” (Stock# 006772), and B6.Cg-Tg(TCRaTCRb)425Cbn/J “OTII” (Stock# 004194) were purchased from Jackson Laboratories; C57BL/6NCrl “CD45.2” and B6.SJL-*Ptprc^a^Pepc^b^*/BoyCrCrl “CD45.1” were purchased from Charles River Laboratories; B6.129S6-*Rag2^tm1Fwa^* “RAG2^−/−^“ were purchased from Taconic; and XCR1-DTR-cre mice were designed and generated by Horizon Discovery using CRISPR-Cas9 (now Inotiv Co, St. Louis, MO) and colony maintained at Charles River Laboratory. Briefly, a mouse codon-optimized T2A-DTR-T2A-Cre cassette was inserted downstream of the Xcr1 coding region by targeting single-cell fertilized embryos with the following sgRNA 5′-tgagagccttcagcaactgg-3′ (**Supplemental Figure 1**). Resulting pups were analyzed by genomic PCR and DNA sequencing to confirm presence of the inserted cassette. Founder mice were outbred to C57BL/6J animals for colony expansion. All animal experiments were conducted in compliance with institutional regulations, under authorized protocols #20221551 and 201401229 approved by the IACUC.

### 2.2 Generation of CAR-T cells and CAR-Tregs

CAR constructs were designed using Geneious software (Dotmatics). The constructs consisted of the mouse (m) CD8 signal peptide fused to the single-chain variable heavy- and light-chain fragments (scFv), mCD28 transmembrane domain, mCD28 intracellular signaling domain, mCD3ζ intracellular signaling domain, internal ribosome entry site (IRES), and mCherry. The scFv regions were anti-mouse XCR1 (clone: ZET), anti-mouse MOG (clone: Z12), and anti-human myostatin (clone: mAb50). The constructs were then synthesized and inserted into a murine stem cell virus (MSCV) vector by GENEWIZ (Azenta Life Sciences).

Retrovirus stocks were generated using a Platinum-E (Plat-E) packaging cell line (Cell Biolabs Inc.) per the manufacturer’s instructions. In brief, Plat-E cells were cultured in DMEM, 1× Pen/Strep, 10 ng/mL blasticidin, 1 pg/mL puromycin (Gibco), and 10% fetal bovine serum (Sigma), transfected using Lipofectamine 3000 (Invitrogen) in antibiotic-free DMEM. After 3-day culture, supernatants were collected and virus was concentrated with Retro-X concentrator (Takara Bio). The concentrated virus was immediately used or aliquoted and frozen at −80°C for later use.

CD3^+^, CD4^+^, and CD8^+^ T cells or CD4^+^ Tregs were purified from the spleens of mice using EasySep Mouse T Cell Isolation Kit, EasySep Mouse CD4^+^ T Cell Isolation Kit, or EasySep Mouse CD8^+^ T Cell Isolation Kit, or EasySep Mouse CD4^+^ CD25^+^ Regulatory T Cell Isolation Kit II, respectively (STEMCELL Technologies). In some cases, Tregs were sorted from FIG mice that express GFP under the FOXP3 promoter. Purified T cells were activated 1:1 or 1:2 with Dynabeads Mouse T-Activator CD3/CD28 (Gibco) and cultured in “T cell media” (RPMI 1640 + GlutaMAX, 1× Pen/Strep, 1× MEM non-essential amino acids, sodium pyruvate (1 nM), HEPES (10 µM), 1× 2-mercaptoethanol (Gibco)), 10% FBS (Sigma), and 3 ng/mL mIL-2 (R&D Systems or PeproTech) overnight. T cells were transduced at 24 and/or 48 h after initial activation with concentrated retrovirus and 4 µg/mL polybrene and centrifuged at 1,200×g for 30 min at 37C. CAR-T cell cultures were moved to fresh T-cell media the following day and maintained at 10^6^ cells/mL, and media were changed every 3–4 days thereafter.

CAR-T cells and CAR-Tregs were not purified before use *in vitro* or *in vivo*, as each transduction gave similar levels of CAR expression, as assessed by mCherry, across groups.

### 2.3 Derivation of bone marrow-derived DC

Bone marrow was collected from the femur and tibia of WT mice and cultured for 10–13 days in IMDM (Gibco) supplemented with GlutaMAX, 1× Pen/Strep, 1× MEM non-essential amino acids, sodium pyruvate (1 nM), 1× 2-mercaptoethanol (Gibco), 10% FBS (Sigma), and 400 ng/mL mFLT3-L (BioLegend).

### 2.4 *In vitro* CAR-T cell and CAR-Treg experiments

RAW264.7 and HEK293T cell lines purchased from ATCC were transfected with XCR1 or MOG construct (synthesized at GENEWIZ), and stably transfected lines were selected with Gentamicin (Gibco). For CAR activation, 100,000 purified CD4^+^ or CD8^+^ XCR1, control, or mock CAR-T cells were cocultured with 50,000 irradiated (60 Gy) RAW, irradiated RAW–XCR1, or bone marrow-derived DC (BMDC) overnight in T-cell media with 3 ng/mL IL-2. For CAR proliferation, 100,000 CellTrace violet (CTV) (Invitrogen)-stained purified CD4^+^ or CD8^+^ XCR1, control, or mock CAR-T cells were cocultured with 50,000 irradiated (60 Gy) RAW, irradiated RAW–XCR1, or BMDC for 3 days in T-cell media with 3 ng/mL IL-2. For CAR-T cell and BMDC cytokine production, 200,000 XCR1, control or mock, CD4^+^, CD8^+^, or CD4^+^ Tregs CAR-T cells were cocultured with 50,000 BMDC for 48 h in T-cell media with 3 ng/mL IL-2. Subsequently, the supernatant was collected and analyzed with a V-PLEX Mouse Cytokine 19-Plex Kit (Meso Scale Diagnostics, LLC) following the manufacturer’s directions. For CAR-Treg activation, 50,000 XCR1, control, or MOG CD4^+^ CAR-Treg cells were cocultured with 50,000 irradiated (30 Gy) RAW, RAW–XCR1, HEK, HEK–MOG, or Dynabeads mouse T-activator beads (Gibco), overnight in complete media with 3 ng/mL IL-2. For CAR-Treg suppression, 25,000 CTV-stained purified CD4^+^ OTII responder T cells were cocultured with 50,000 irradiated (30 Gy) BMDC and 50,000 RAW, RAW–XCR1, or RAW–MOG cells in complete media with 64nM OVA (323–339) peptide. XCR1 or MOG CAR-Tregs were titrated and added at designated ratios.

### 2.5 *Ex-vivo* LPS stimulation of splenocytes

Spleens were dissected from CD4^+^ or CD8^+^ XCR1 CAR-T cell-treated mice and placed into media containing 2 mg/mL collagenase D (Roche) and RNase-free DNase (Qiagen), cut into small pieces, incubated at 37°C for 30–60 min under agitation, filtered over a 70-μm cell strainer (Corning, NY), and washed with wash buffer (PBS + 1 mM EDTA + 2% FBS) to obtain a single-cell suspension. RBCs were lysed with 5 mL of ACK lysis buffer (Gibco) for 5 min and neutralized with wash buffer. Next, a quarter of the total splenocytes were resuspended in T cell media with 10 μg/mL LPS for 24 h. Then, the supernatant was collected subsequently analyzed with a V-PLEX Mouse IL-12p70 Kit (Meso Scale Diagnostics, LLC) following the manufacturer’s directions.

### 2.6 Tissue processing and flow cytometry

The inguinal lymph nodes (ILN), cervical lymph nodes (CLN), spleen, liver, lung, and/or CNS were dissected from mice and placed into media containing 2 mg/mL collagenase (Roche) and RNase-free DNase (Qiagen), cut into small pieces, incubated at 37°C for 30–60 min under agitation, filtered over a 70-mm cell strainer (Corning, NY), and washed with wash buffer (PBS+ 1 mM EDTA + 2% FBS) to obtain a single-cell suspensions. For the spleen, RBCs were lysed with 5 mL of ACK lysis buffer for 5 min and neutralized with wash buffer. CNS, liver, and lung cells were further enriched using Percoll gradient. Protocol was adapted from the following (36–40). In short, cells were resuspended in 6 mL of 37% Percoll, overlaid on top of 6 mL of 70% Percoll, and centrifuged at 2,000 RPM for 20 min with no break at 4°C. The interphase containing the enriched leukocytes was collected and use.

Antibodies used for flow cytometric analysis are listed in **Supplemental Table 1**. Single-cell suspensions were blocked with Fc-block for 10 min and then surface stained with cocktails of fluorochrome-conjugated antibodies in brilliant stain buffer (BD) with 1:1,000 LIVE/DEAD™ Fixable Near-IR Dead Cell Stain kit (Invitrogen) for 30 min. For intracellular staining, samples were fixed and permeabilized and stained with Foxp3/Transcription Factor Staining Buffer Set (eBioscience) per manufacturer’s directions. To obtain cell counts, CountBright™ Plus Absolute Counting Beads (Invitrogen) were added to samples immediately before flow cytometric analysis. Samples were acquired on Symphony Cytometer (BD) using BD FACSDiva software and then analyzed using FlowJo software.

### 2.7 Induction and assessment of EAE

For active induction of EAE, mice were subcutaneously (sc) immunized with 200 µL of MOG^35-55^ (1 mg/mL) in CFA emulsion (Hooke Laboratories) at two separate sites (100 µL/right and left hind flank). Subsequently, mice were IP injected with 100–120 ng of pertussis toxin (PTx) (Hooke Laboratories) in saline on days 0 and 2. For passive EAE, donor mice (WT) were immunized as stated above with MOG^35-55^/CFA, but without PTx injection. Then, 10–12 days post-immunization, spleen and ILN were harvested, processed into single-cell suspension, and reactivated in T cell media supplemented with 10 µg/mL MOG^35-55^, 20 ng/mL IL-12, and 10 µg/mL anti-IFN-γ (clone XMG1.2) for 72 h. Then, CD3^+^ or CD4^+^ T cells were purified from cultures with EasySep Mouse T Cell Isolation Kit or EasySep Mouse CD4^+^ T Cell Isolation Kit (STEMCELL Technologies) per the manufacturer’s instructions. On day 0, gender-matched recipients (RAG2^−/−^, XCR1-DTR, or CD57BL/6 mice) were intravenously (iv) injected with purified encephalitogenic CD3^+^ or CD4^+^ T cells in 100 μL of saline. Subsequently, recipient mice were injected IP with 100–120 ng of PTx (Hooke Laboratories) in saline on days 0 and 2. Mice were monitored daily for clinical EAE score and body weight. The EAE score was determined by the following clinical scale: 0—asymptomatic; 0.5—partial limp tail or body tremors; 1—limp tail but normal gait; 2—limp tail and hind limb weakness (waddle); 3—partial hind limb paralysis; 4—full hind limb paralysis; 5—severe lack of mobility due to forelimb paralysis or lack of interest in food/water or severe weakness and the humane endpoint. To calculate the percent change in body weight, 100% body weight was assigned at day 0; daily weights thereafter were normalized to this starting value. The mean clinical EAE score was the average EAE score for each mouse across the defined period. When mice reached a humane endpoint, they were scored as a 5 and excluded from the mean clinical EAE score and percent change in body weight thereafter.

### 2.8 Statistical analysis

All graphed data are presented with the mean and standard error of the mean (SEM). Statistical significance for the mean clinical EAE scores was analyzed with a Friedman test followed by Dunn’s multiple comparisons test for experiments with three plus groups or a Wilcoxon-matched pair test for experiments with two groups. Mean percent change in body weight was analyzed with a two-way repeated-measures ANOVA followed by Šídák multiple comparison test. The percent EAE incidents were analyzed with Gehan–Breslow–Wilcoxon test. All other data containing three or more groups were analyzed with one-way ANOVA with Tukey’s multiple comparison test, and data containing two groups were analyzed with unpaired two-tailed t test. Statistical analysis was performed using GraphPad Prism 7 (GraphPad Software, La Jolla, CA), and a p-value of 0.05 or less was considered statistically significant.

## 3 Results

### 3.1 Design, generation, and expression of XCR1 CAR construct in T cells and Tregs

**Figure 1:**
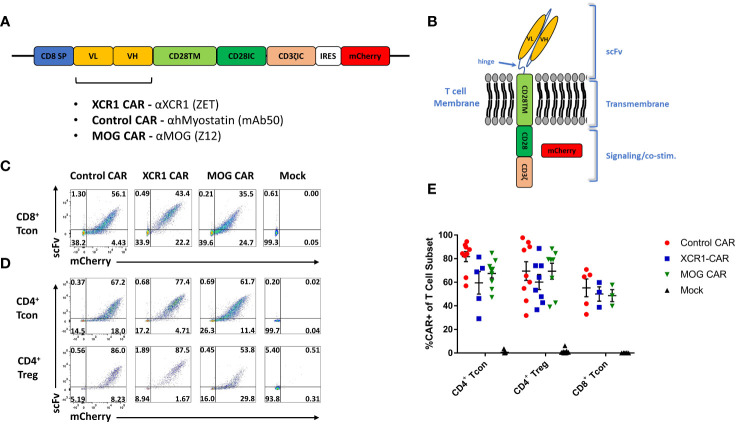
Design, generation, and expression of XCR1 CAR-T cells and CAR-Tregs. Shown **(A)** is a schematic of the CAR construct and **(B)** a schematic of the CAR embedded in a cell membrane. **(C-E)** CD8^+^ T cell, CD4^+^ T cell, or CD4^+^ Tregs were purified from spleens of C57BL/6 or FOXP3-GFP mice, activated with Dynabeads Mouse T-Activator CD3/CD28 beads + 3 ng/mL IL-2 in T cell media and transduced with XCR1, control, MOG, or mock CAR constructs at 24 and/or 48 h Shown are representative dot plots of surface CAR (y-axis) and mCherry (x-axis) of **(C)** CD8^+^ T cells on day 6 and **(D)** CD4^+^ T cell (top) or CD4^+^ Tregs (bottom) on day 5 post activation. Graphed **(E)** are the %mCherry^+^ CAR-T cells for CD4^+^ T cell, CD4^+^ Treg, and CD8+ T cell between days 2 and 9 post transfection from 16 pooled individual experiments. Error bars represent SEM.

We aimed at developing a novel CAR targeting DC1 as they are thought to constitute an inflammatory DC subset and are major contributors of the pathogenic cytokine IL-12. For that purpose, DC1 were targeted *via* the DC1-specific extracellular XCR1. A second-generation CAR construct consisting of an extracellular recognition domain and intracellular CD3ζ and CD28 signaling domains was selected. The CD28 intracellular signaling domain was chosen because of its well-characterized role in CAR-T cell and CAR-Treg function (41, 42) and utilization in clinical CAR-T cell constructs (TECARTUS and YESCARTA). In all, the DC1-targeted “XCR1 CAR” construct consisted of the mouse (m)CD8 signal peptide fused to the anti-XCR1 single-chain variable fragment heavy and light chains (scFv), the mCD28 transmembrane domain, the mCD28 intracellular signaling domain, the mCD3ζ intracellular signaling domain, the internal ribosome entry site (IRES), and mCherry (**Figures 1A, B**). We also generated “MOG” CAR constructs with anti-myelin oligodendrocyte protein (MOG) scFv and “control” anti-human myostatin scFv as a non-specific CAR. Expression of mCherry was used as a surrogate for CAR expression.

Purified CD4^+^ T cells, CD8^+^ T cells, or FOXP3^+^ CD4^+^ Tregs were transduced, and surface expressions of XCR1, MOG, and control CAR were confirmed with co-staining of anti-IgG-AF647, which bound to the scFv, and mCherry (**Figures 1C, D**). All constructs were successfully transduced, and CAR constructs were highly expressed on the surface of CAR-T cells and CAR-Tregs. We compiled CAR expression (days 2–9) over multiple independent experiments and found that transduction efficiency was between ~30% and 85% for XCR1, MOG, and control CAR-T cells and CAR-Tregs (**Figure 1E**).

### 3.2 CD4^+^ and CD8^+^ XCR1 CAR-T cells are functionally active *in vitro*

**Figure 2:**
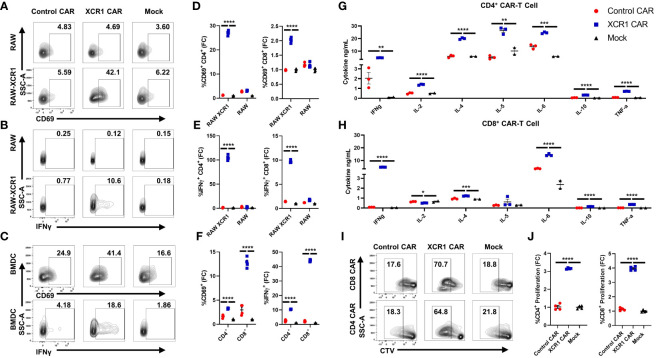
CD4^+^ and CD8^+^ XCR1 CAR-T cells are functionally active *in vitro*. **(A-F)** 100,000 XCR1, control, or mock, CD4^+^ or CD8^+^, CAR-T cells were cocultured with 50,000 irradiated RAW, irradiated RAW–XCR1, or BMDC overnight in complete media with 3 ng/mL IL-2. **(A-F)** CAR-T cells were assessed for activation markers *via* flow cytometry. Shown **(A-C)** are representational dot plots of CD4^+^ CAR-T cells assessed for **(A)** SSC-A (y-axis) and CD69 (x-axis), **(B)** SSC-A (y-axis) and IFN-γ (x-axis), **(C)** SSC-A (y-axis) and CD69 (x-axis, top) and IFN-γ, (x-axis, bottom) after coculture with **(A, B)** RAW–XCR1 or RAW and **(C)** BMDC. Graphed are the fold change (FC) of **(D)** %CD69^+^ and **(E)** %IFN-γ^+^ for CD4^+^ (left) or CD8^+^ (right) CAR-T cell cultures cocultured with RAW–XCR1 or RAW. Graphed **(F)** are the fold change of %CD69^+^ (left) and %IFN-γ^+^ (right) CD4^+^ or CD8^+^ CAR-T cell cultures cocultured with BMDC. 200,000 XCR1, control, or mock **(G)** CD4^+^ or **(H)** CD8^+^ CAR-T were cocultured with 50,000 BMDC for 48 h in T-cell media with 3 ng/mL IL-2. Subsequently, the supernatant was analyzed for cytokine concentration. Graphed are concentrations of INF-γ, IL-2, IL-4, IL-5, IL-6, IL-10, and TNF-α ng/mL for each treatment group. **(I, J)** 100,000 cell trace violate (CTV)-stained XCR1, control, or mock; CD4^+^ or CD8^+^, CAR T cells were cocultured with 50,000 BMDC for 3 days in complete media with 3 ng/mL IL-2. Shown **(I)** are representational dot plots of CD8^+^ (top) or CD4^+^ (bottom) CAR T cells assessed for SSC-A (y-axis) and CTV (x-axis). Graphed **(J)** are the fold change %proliferating (CTV dye diluted) of CD4^+^ (left) or CD8^+^ (right) CAR-T cultured with BMDC. Fold change was calculated by dividing the %CAR positive population by the mean % positive population of mock CAR T cells. Statistical significance was analyzed by use of one-way ANOVA and Tukey’s multiple comparisons test *p < 0.05, **p < 0.01 ***p < 0.001, ***p < 0.001. Error bars represent SEM. Data are representative of two independent experiments.

To test the *in vitro* function of XCR1 CAR-T cells (**Figure 2**), XCR1, control or mock, CD4^+^ or CD8^+^, CAR-T cells were cocultured with either irradiated RAW or irradiated RAW cells stably transduced with a XCR1 construct (RAW–XCR1) or bone marrow-derived dendritic cells (BMDC) (**Figures 2A–F**). The CAR-T cells were assessed for CD69, IFN-γ, CD25, and IL-2 expression *via* flow cytometric analysis. Both CD4^+^ and CD8^+^ XCR1 CAR-T cells exhibited increased frequencies of CD69 (**Figures 2A, D**), IFN-γ (**Figures 2B, E**), CD25 (**Supplemental Figure 2A, B**), and IL-2 (**Supplemental Figure 2C, D**) positive cells in response to RAW–XCR1 cells as compared with control and mock CAR-T cells. When cocultured with the XCR1^−^ RAW cell line, XCR1, control, and mock CAR-T cells expressed similar levels of CD69, IFN-γ, CD25, and IL-2 (**Figures 2A–E**; **Supplemental Figures 2A–D**). XCR1 CAR-T cells were also specifically activated in response to BMDC, which endogenously express XCR1, because XCR1 CAR-T cells exhibited increased frequencies of CD69 (**Figures 2C, F**), IFN-γ (**Figures 2C, F**), CD25 (**Supplemental Figure 2E**), and IL-2 (**Supplemental Figure 2F**) positive cells compared with control or mock CAR-T cells. Therefore, CD4^+^ and CD8^+^ XCR1 CAR-T cells specifically responded to XCR1^+^ but not XCR1^—^ cell lines.

Next, we characterized the cytokine expression profiles of CD4^+^ and CD8^+^ CAR-T cells in coculture with BMDC, which can also produce cytokines in response to T cells, *in vitro*. XCR1, control, or mock, CD4^+^ or CD8^+^ CAR-T cells or CD4^+^ CAR-Tregs were cocultured with BMDC for 48 h, and then supernatant was collected and cytokine production was analyzed via Meso Scale Discovery (MSD) immunoassay (**Figures 2G, H**; **Supplemental Figures 3A–O**). CD4^+^ and CD8^+^ XCR1 CAR-T and BMDC coculture produced significant amounts of IFN-γ, IL-4, IL-6, IL-10, and TNF-α (**Figures 2G, H**) compared with mock and control CAR-T cells. However, only CD4^+^ XCR1 CAR-T cells produced significant levels of IL-2 and IL-5. In addition, CD4^+^ XCR1-CAR T cells produced higher levels of potentially proinflammatory cytokines IL-2, IL-4, IL-5, IL-6, and TNF-a in comparison with CD8^+^ XCR1 CAR-T cells. CD8^+^ XCR1 CAR-T cells and BMDC coculture produced significantly more chemokines including MIP-1 and IP-10 and exhibited increased IL-1β compared with CD4^+^ XCR1 CAR T cells (**Supplemental Figures 3H, I, L**). Mock and control CAR-T cells and BMDC coculture produced similar levels of each cytokine (**Figures 2G, H**; **Supplemental Figures 3A–O**). Together, these data show that both CD4^+^ and CD8^+^ XCR1 CAR-T cells respond to DCs and exhibit unique cytokine profiles, in which CD4^+^ CAR-T cells make higher amounts of potentially pathogenic cytokines in comparison with CD8^+^ CAR-T cells.

To determine if XCR1 CAR-T cells can proliferate in response to XCR1^+^ BMDC, we cocultured CTV-stained CD4^+^ or CD8^+^ CAR-T cells with BMDC for 3 days. XCR1 CAR-T cells exhibited increased proliferation, determined by CTV dilution, when cocultured with BMDC (**Figures 2I, J**) compared with control and mock CAR-T cells. Overall, these results showed that the XCR1-specific CAR engendered CD4^+^ and CD8^+^ T cells with XCR1-dependent effector functions including cytokine production and ability to proliferate.

### 3.3 XCR1 CAR-T cells transiently persist but fail to deplete DC1 in immunocompetent mice

**Figure 3:**
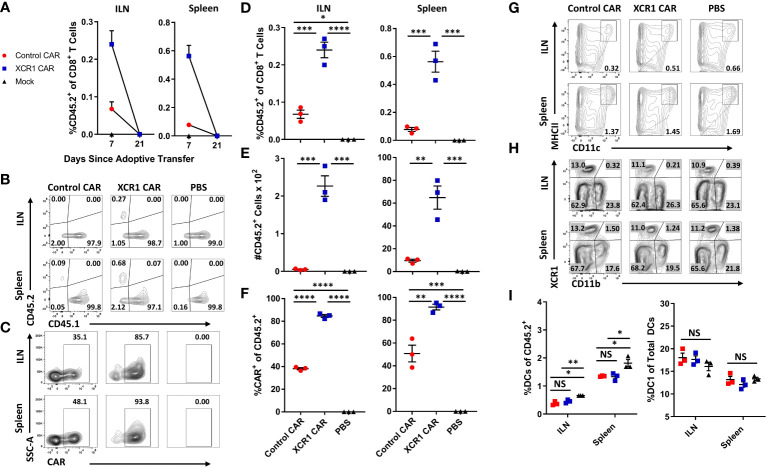
XCR1 CAR-T cells transiently persist but fail to deplete DC1 in immunocompetent mice. **(A-I)** CD45.1 mice were iv injected with PBS, 1 x 10^6^ XCR1, or control CD45.2^+^ CD8^+^ CAR T cells (n = 3/group) and 10 µg of IL-2 sc on day 0. On days 7 and 21, ILN and spleen were collected from treated mice and analyzed *via* flow cytometric analysis for CAR-T cells and DC subsets. Graphed **(A)** are the percent CD45.2^+^ cell of CD8^+^ T cells on days 7 and 21 for the ILN (left) and spleen (right). Shown **(B)** are representational dot plots of CD8^+^ T cells assessed for CD45.1 (x-axis) and CD45.2 (y-axis) and **(C)** CD45.2^+^ cells assessed for SSC-A (y-axis) and mCherry (x-axis) from the ILN (top) and spleen (bottom) on day 7. The percent **(D)** and number **(E)** of CD45.2^+^ cells of CD8^+^ T cells and **(F)** percent CAR^+^ T cells of CD45.2^+^ donor cells on day 7 for the inguinal LN (left) and spleen (right). Shown **(G)** are representational dot plots of viable cells assessed for SSC-A (y-axis) and CD11c (x-axis) and **(H)** DCs (CD11c^+^ MHCII^+^) assessed for XCR1 (y-axis) and CD11b (x-axis) from the ILN (top) and spleen (bottom). Graphed **(I)** are the percent DCs (MHCII^+^ CD11c^+^) of viable cells (left) and percent DC1 of total DCs (right) in the ILN and spleen. Statistical significance was analyzed by use of one-way ANOVA Tukey’s multiple comparisons test. *p < 0.05, **p < 0.01, ***p < 0.001, ****p < 0.0001. Error bars represent SEM. Data are representative of two independent similar experiments.

To test the *in vivo* function of XCR CAR-T cells, WT CD45.1^+^ mice (n = 3/group) were iv injected with either PBS, 1 x 10^6^ CD45.2^+^ CD8^+^ XCR1, or 1 x 10^6^ CD45.2^+^ CD8^+^ control CAR-T alongside 10 µg of IL-2 sc to promote T-cell engraftment (**Figure 3**). The inguinal lymph node (ILN) and spleen were harvested and analyzed by flow cytometry for CAR-T cells and DC subsets on days 7 and 21. XCR1 CAR-T cells were recovered from the ILN and spleen on day 7 but were undetectable by day 21 (**Figure 3A**). On day 7, XCR1 CAR-T cells made up ~0.24% and ~0.56% and control CAR-T made up ~0.07% and ~0.1% of the total CD8^+^ T cells in the lymph nodes and spleen, respectively (**Figures 3B, D**). Likewise, there were significantly more XCR1, as a number, than control CAR-T cells in the ILN and spleen (**Figure 3E**). In addition, XCR1 CAR-T cells were enriched in mCherry^+^ (CAR^+^) cells as 85%–92% of the CD45.2^+^ donor cells were mCherry^+^ compared with 38%–50% of control CAR-T cell-treated mice (**Figures 3C, F**). While XCR1 CAR-T cells persisted until day 7, they failed to deplete XCR1^+^ DC1 cells. Both the percent DC (MHCII^+^ CD11c^+^) of viable cells (**Figures 3G, I**) and the percent DC1 (XCR1^+^ CD11b^—^) of total DC (**Figures 3H, I**) were unchanged between XCR1 and control CAR-T cell-treated mice. Interestingly, both XCR1 and control CAR-T cell treatment led to some decrease in DC frequencies as compared with PBS-treated mice (**Figure 3I**). To determine if CD4^+^ CAR-T cells were required for successful engraftment and DC1 depletion, we treated CD45.2^+^ mice with 10 × 10^6^ CD45.1^+^ CD3^+^ (mixed CD4/CD8) XCR1 CAR-T cells and on day 8 CAR-T and DC populations were analyzed (**Supplemental Figures 4A–E**). As seen with CD8^+^ CAR T cells alone the XCR1 CD3^+^ CAR-T cells persisted and failed to deplete DC1. In all, we concluded that XCR1 CAR-T cells transiently persist but fail to deplete DC1.

### 3.4 XCR1 CAR-T cells engraft and deplete DC1 in immunodeficient mice

**Figure 4:**
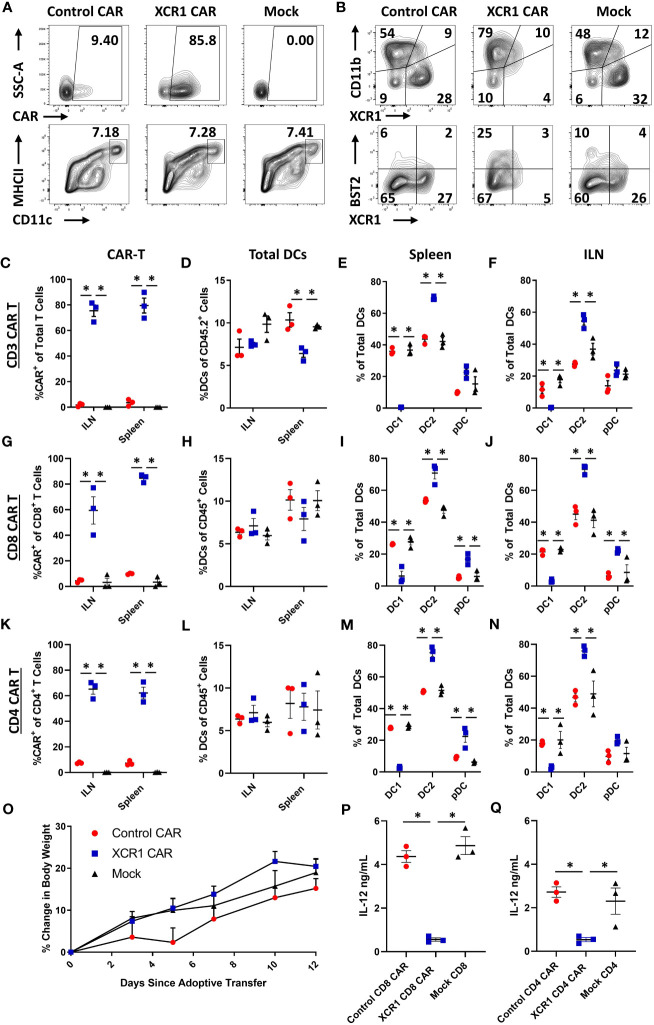
XCR1 CAR-T cells engraft and deplete DC1 in immunodeficient mice. RAG2^−/−^ mice were iv injected with 1 x 10^6^ XCR1, control, or mock transduced **(A, B, G–J, P)** CD8^+^, (**C-F, O**) CD3^+^ (+ 10 μg IL-2), or **(K–N, Q)** CD4^+^ T cells on day 0 (n = 3/group). On day 7 or 12, spleen and ILN were collected and analyzed *via* flow cytometric analysis for CAR-T and DC subsets. Shown **(A)** are representational dot plots of the following: row 1, donor T cells (TCRβ^+^ CD8^+^) assessed for SSC-A (y-axis) and mCherry (x-axis); row 2, recipient CD45^+^ cells for MHCII (y-axis) and CD11c (x-axis). Shown **(B)** are representational dot plots of recipient DCs (MHCII^+^ CD11c^+^) for CD11b (y-axis) and XCR1 (x-axis), and DCs for BST2 (y-axis) and XCR1 (x-axis) from the spleen of mice treated with CD8^+^ CAR-T cells. Graphed are the percent CAR^+^ (mCherry^+^) of the **(C)** TCRβ^+^ **(G)**, CD8^+^, and **(K)** CD4^+^ T cells and **(D, H, L)** the percent DCs (MHCII^+^ CD11c^+^) of total CD45^+^ from the ILN and spleen of mice treated with **(C, D)** CD3^+^ **(G, H)**, CD8^+^, and **(K, L)** CD4^+^ CAR-T cells. The percent DC1 (XCR1^+^ CD11b^−^), DC2 (XCR1^−^ CD11b^+^), and pDC (XCR1^−^ BST2^+^) of total DC are shown for the spleen **(E, I, M)** and the ILN **(F, J, N)** for mice treated with **(E, F)** CD3^+^, **(I, J)** CD8^+^, and **(M, N)** CD4^+^ CAR-T cells. Graphed **(O)** is the Percent change in body weight from day 0 to the end of the experiment on day 12 of mice treated with CD3^+^ CAR-T cells. On day 7, a quarter of the total splenocytes were restimulated with 10 μg/mL LPS in T cell media for 24 h, and then ng/mL IL-12 was determined *via* MSD. Shown **(P, Q)** are ng/mL of IL-12 from **(P)** CD8^+^ or **(O)** CD4^+^ CAR-T cell-treated mice. Statistical significance was analyzed by use of one-way ANOVA Tukey’s multiple comparisons test. *p < 0.05. Error bars represent SEM. Data **(A–J)** are representative of three independent similar experiments.

Because CAR-T cells failed to durably engraft or mediate effector functions in immunocompetent mice, we tested whether CD3^+^, CD8^+^, or CD4^+^ XCR1 CAR-T cells could engraft and mediate DC1 depletion in immunodeficient RAG2^−/−^ mice (**Figure 4**). RAG2^−/−^ mice (n = 3/group) were iv injected with 1 x 10^6^ XCR1, control, or mock-transduced CD3^+^ (with 10 μg of IL-2), CD8^+^, or CD4^+^ XCR1 CAR-T cells. Mice treated with CD3^+^ CAR-T cells were given IL-2 to ensure T-cell engraftment, whereas mice treated with CD4^+^ or CD8^+^ CAR-T cells were not. On day 7 or 12, ILN and spleen were harvested and analyzed by flow cytometric analysis for the presence of CAR-T cells and DC subsets. CD3^+^, CD8^+^, and CD4^+^ XCR1 CAR-T cells successfully engrafted in RAG2^−/−^ mice in comparison with control and mock CAR-T cells (**Figures 4A, C, G, K**). Specifically, ~40%–95% of donor cells were CAR^+^ in CD3^+^, CD8^+^, and CD4^+^ XCR1 CAR-T cell-treated mice in comparison with an average of ~1%–15% or less for control and mock CAR-T cell-treated mice. Engraftment of CD3^+^, CD8^+^, and CD4^+^ XCR1 CAR-T cells did not change the overall frequencies of DC (**Figures 4D, H, L**) except for a slight decrease in the DC frequencies within total viable cells in the spleen of CD3^+^ XCR1 CAR-T cells treated mice compared with control and mock CAR-T cell-treated mice (**Figure 4D**). Moreover, CD3^+^, CD8^+^, and CD4^+^ XCR1 CAR-T cells specifically and almost entirely depleted DC1 (XCR1^+^ CD11b^−^) in the ILN and spleen of treated mice (**Figures 4E, F, I, J, M, N**). For example, DC1 comprised less than 1% of the total DC in the spleen of CD3^+^ XCR1 CAR-T cell-treated mice, compared with ~30% in control and mock CD45.1^+^ CAR-T cell-treated mice (**Figure 4E**). DC1 depletion resulted in compensatory increases in the percentages of DC2 (XCR1^−^ CD11b^+^) in the spleen and ILN of CD3^+^, CD8^+^, and CD4^+^ XCR1 CAR-T cell-transferred mice compared with control and mock CAR-T cell-treated mice (**Figures 4E, F, I, J, M, N**). Likewise, pDC (XCR1^−^ BST2^+^) of total DCs were increased in the spleens of CD8^+^ and CD4^+^ XCR1 CAR-T cell-treated mice compared with control and mock CAR-T cell-treated mice (**Figures 4I, M**). Thus, XCR1 CAR-T cells could efficiently deplete DC1 leading to overrepresentation of DC2 and pDC among total DC. In addition, IL-2 was not required for adequate engraftment of either CD4^+^ or CD8^+^ CAR-T cells.

To determine if CAR-T cell engraftment led to elevated cytokine levels *in vivo*, serum was collected on day 7 from RAG2^−/−^ mice engrafted with CD8^+^ or CD4^+^ XCR1 or control CAR-T cells (**Supplemental Figures 5A–D**). Mice treated with CD4^+^ XCR1 CAR-T cells had increased levels of TNF-α, IL-27, IL-10, and IP-10 compared with mice treated with control CAR-T and CD8^+^ XCR1 CAR T cells. Other tested cytokines were either unchanged compared with control CAR-T cells or undetectable. This suggested that CD4^+^ XCR1 CAR-T cells produce proinflammatory cytokines *in vivo* following engraftment.

To determine if CAR-T cell engraftment led to activation of DC subsets, the ILN, spleen, and lung were collected on day 7 from RAG2^−/−^ mice treated with CD8^+^ and CD4^+^ XCR1, control, or mock CAR-T cells (**Supplemental Figures 6A–F**). Both CD8^+^ and CD4^+^ XCR1 CAR-T cell engraftment led to increased expression of activation markers CD80, CD86, PD-L1, and CD40 on DC2 and pDC in the ILN of treated mice. However, DCs in the lung were unchanged. Therefore CAR-T cell engraftment leads to DC activation in the ILN of treated mice.

Mice were periodically weighed and monitored to determine if engraftment of self-reactive CD3^+^ XCR1 CAR-T cells resulted in overt autoimmunity. Throughout the 12-day experiment, mice did not exhibit detectable weight loss but gained weight at similar rates across all three groups (**Figure 4O**). Likewise, mice were active, alert, and without notable physical signs of distress. Together, these results suggest that XCR1 CAR-T cells engraft in immunodeficient RAG2^−/−^ mice and eliminate DC1 in lymphoid organs without acute autoimmunity.

To determine if DC1 depletion resulted in decreased IL-12 production, spleens from CD4^+^ and CD8^+^ XCR1 CAR-T cell-treated mice were processed into single-cell suspensions and restimulated *ex-vivo* with LPS for 24 h, and subsequently the IL-12 concentration was determined *via* MSD (**Figures 4P, Q**). Spleens from mice treated with CD4^+^ and CD8^+^ XCR1 CAR-T cells, which were depleted of DC1, produced significantly reduced levels of IL-12 compared with spleens from mice treated with control or mock CAR-T cells. Therefore, DC1 depletion reduced the production of IL-12 in the spleen.

### 3.5 XCR1 CAR-T cells systemically deplete DC1 when engrafted with encephalitogenic T cells in immunodeficient mice

**Figure 5:**
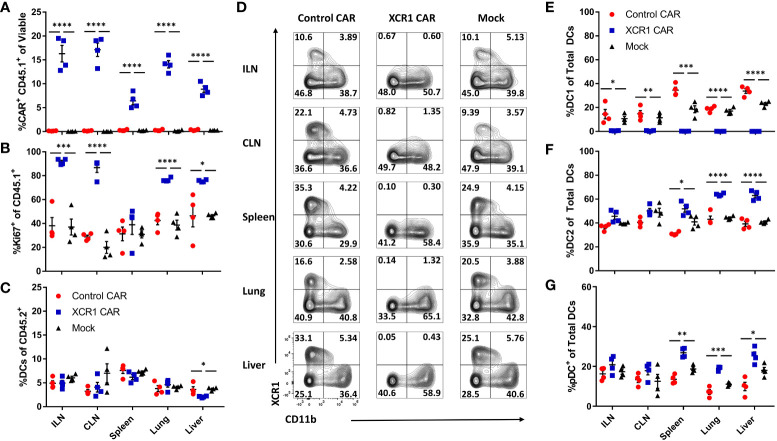
XCR1 CAR-T cells systemically deplete DC1 when engrafted with encephalitogenic T cells in immunodeficient mice. RAG2^−/−^ mice (n = 4/group) were iv injected with 1 x 10^6^ XCR1, control, or mock transduced CD45.1^+^ CD8^+^ CAR-T alongside 1 x 10^6^ encephalitogenic CD4^+^ CD45.2^+^ T cells and 10 µg of IL-2 IP on day 0. On day 6, ILN, CLN, spleen, lung, and liver were collected and analyzed *via* flow cytometric analysis for CAR-T cells and DC subsets. Shown **(A)** the percent CAR^+^ (mCherry^+^) CD45.1^+^ of viable and **(B)** the percent Ki67^+^ of donor CD45.1^+^ cells and **(C)** the percent DC of total CD45.2^+^. Shown **(D)** are representational dot plots of DC (MHCII^+^ CD11c^+^) for XCR1 (y-axis) and CD11b (x-axis) for each organ. Graphed are **(E)** the percent DC1 (XCR1^+^ CD11b^−^), **(F)** DC2 (XCR1^−^ CD11b^+^), and **(G)** pDC (XCR1^−^ BST2^+^) of total DCs. Statistical significance was analyzed by use of one-way ANOVA Tukey’s multiple comparisons test. *p < 0.05, **p < 0.01, ***p < 0.001, ****p < 0.0001. Error bars represent SEM.

As CD3^+^, CD8^+^, and CD4^+^ XCR1 CAR-T cells engrafted and depleted DC1 in RAG2^−/−^ mice, we sought to determine if CD8^+^ CAR-T cells could systemically deplete DC1 (**Figure 5**). Therefore, 1 x 10^6^ CD45.1^+^ CD8^+^ XCR1, control, or mock CAR-T cells and 1 x 10^6^ encephalitogenic CD45.2^+^ CD4^+^ T cells derived from MOG^35-55^/CFA-immunized donors were iv injected into RAG2^−/−^ mice along with 10 µg of IL-2 via Intraperitoneal (IP) injection (n = 4/group). On day 6, the ILN, cervical lymph nodes (CLN), spleen, liver, and lung were harvested and analyzed *via* flow cytometry for CAR-T cells and DC subsets. XCR1 CAR-T cells engrafted in all the analyzed organs such that ~5%–17% of the total recovered viable cells were CD45.1^+^ mCherry^+^ (CAR^+^) compared with less than 0.5% for control and mock CAR-T cell-treated mice, respectively (**Figure 5A**). In addition, the frequency of Ki67^+^ cells was increased among XCR1 CAR-T cells, indicating *in vivo* antigen recognition, activation, and proliferation in most of the organs analyzed (**Figure 5B**). Encephalitogenic CD45.2^+^ CD4^+^ cells engrafted alongside CD45.1^+^ CD8^+^ CAR-T cells as there were similar percentages of TCRβ^+^ T cells out of the total CD45.2^+^ cells in the organs analyzed among each group (**Supplemental Figure 7A**).

XCR1 CAR-T cells did not significantly alter the percentage of DC in the ILN, CLN, spleen, or lung from host mice (CD45.2^+^) compared with control and mock CAR-T cell-treated mice (**Figure 5C**). However, there was a slight decrease (1.8-fold) in frequencies of DCs in the liver of XCR1 CAR-T cells compared with control and mock CAR-T cell-treated mice. Impressively, XCR1 CAR-T cells systemically depleted DC1 in all organs analyzed, such that less than 1% of DC were DC1 in the ILN, CLN, spleen, lung, and liver compared with 15%–35% in control and mock CAR-T cell-treated mice (**Figures 5D, E**). Moreover, DC1 depletion led to a systemic increase in the frequencies of DC2 (**Figure 5F**) and pDC (**Figure 5G**) in the spleen, liver, and lung of XCR1 CAR-T-treated mice. Together, these results showed that CD8^+^ XCR1 CAR-T cells mediate systemic DC1 depletion without leading to systemic changes in total DC frequencies. In addition, both XCR1 CAR-T cells and encephalitogenic donor CD4^+^ T cells can concurrently engraft in RAG2^−/−^ recipients, allowing the prospect of testing XCR1 CAR-T cells in adoptive transfer EAE in RAG2^−/−^ mice.

Numerous cell surface markers including CD8 and CD103 have been used to identify DC1 populations *in vivo*. Therefore, we assessed CD8^+^ XCR1 CAR-T cells ability to deplete CD8^+^ and CD103^+^ “DC1” subsets in the spleen, lymph nodes, and CNS (**Supplemental Figure 8**). We determined that XCR1 CAR-T cells specifically depleted XCR1^+^ cells and spared CD8 and CD103 single positive DC1. Interestingly, CD103 single positive DC1 make up the majority of the “DC1” in the CNS, whereas the majority of “DC1” in the spleen and ILN are XCR1^+^. Therefore, XCR1 CAR-T cells have a major impact on DC1 in the spleen and lymph nodes while having a more subtle effect in the CNS. In all, the XCR1 CAR T cell only depletes DC1 populations that express XCR1 while sparing other XCR1^−^ DC1 subsets.

### 3.6 XCR1 CAR-T cells suppressed the onset of CD4^+^ T cell-induced passive EAE

**Figure 6:**
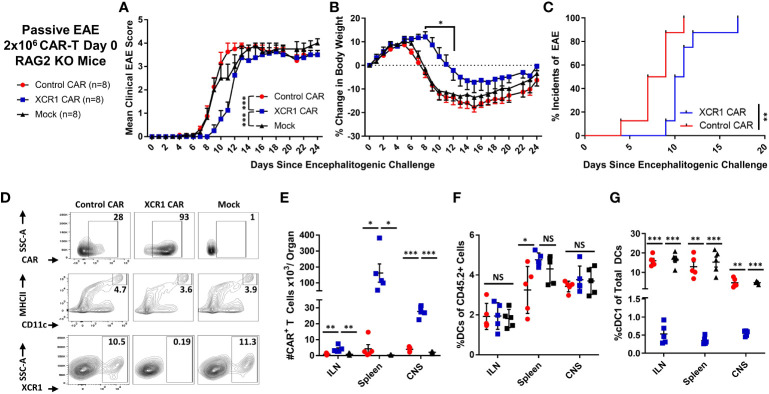
XCR1 CAR-T cells suppressed the onset of CD4^+^ T cell-induced passive EAE. **(A-G)** EAE was induced in RAG2^−/−^ mice by iv injection with 2 × 10^6^ CD45.2^+^ CD4^+^ encephalitogenic T cells followed by PTx on days 0 and 2. In addition, mice were treated with 2 × 10^6^ XCR1, control, or mock CD45.1^+^ CD8^+^ CAR-T (n = 8/group) and 10 µg of IL-2. Shown **(A)** are the mean clinical EAE scores and **(B)** the mean percent change in body weight from day 0 through the end of the experiment on day 24 for each group. Shown **(C)** is the initial day of EAE disease onset from day 0 through day 24 for XCR1 and control CAR-T treatment groups. Spleen, ILN, and CNS were harvested from five mice from each group on day 24 and analyzed *via* flow cytometry for presence of CAR-T cells and DC subset. Shown **(D)** are representative dot plots of the following: first row, CD45.1^+^ donor cells assessed for mCherry; second row, CD45.2^+^ cells assessed for MHCII (y-axis) and CD11c (x-axis); and third row, DC (CD11c^+^ MHCII^+^) assessed for SSC-A (y-axis) and XCR1 (X-axis) from the spleen. The percent **(E)** CAR^+^ (mCherry^+^) T cells, **(F)** percent DC (CD11c^+^ MHCII^+^) of the CD45.2^+^ cells, and **(G)** percent DC1 (XCR1^+^) of the total CD45.2^+^ DC are shown for each organ on day 24. Statistical significance was analyzed by use of **(A)** Friedman test followed by Dunn’s multiple comparisons test, **(B)** two-way repeated measures ANOVA followed by Šídák multiple comparison test, **(C)** Gehan–Breslow–Wilcoxon test, **(E–G)** one-way ANOVA Tukey’s multiple comparisons test. *p < 0.05, **p < 0.01, ***p < 0.001. Error bars represent SEM. Data are representative of two independent experiments.

A murine model of EAE was used to determine if DC1 depletion with CD8^+^ XCR1 CAR-T cells inhibited the development of neuroinflammation (**Figure 6**). CD8^+^ CAR-T cells were utilized because they produced less proinflammatory cytokines as compared with CD4^+^ CAR-T cells. Because CD8^+^ XCR1 CAR-T cells required a lymphopenic host for engraftment, we utilized a passive EAE model, in which encephalitogenic CD4^+^ T cells were transferred into RAG2^−/−^ mice. On day 0, 2 × 10^6^ encephalitogenic CD45.2^+^ CD4^+^ T cells and 2 × 10^6^ XCR1, control, or mock transduced CD45.1^+^ CD8^+^ CAR-T cells alongside 10 µg of IL-2 were iv injected into RAG2^−/−^ recipients (n = 8/group). Subsequently, RAG2^−/−^ mice were IP injected with (PTx) on days 0 and 2.

As expected, RAG2^−/−^ mice treated with encephalitogenic T cells and mock CAR-T cells developed severe EAE (**Figures 6A, B**). However, RAG2^−/−^ mice treated with XCR1 CAR-T cells exhibited delayed EAE onset (**Figures 6A–C**) as measured by significantly reduced mean clinical EAE scores (**Figure 6A**) and increased body weight on days 9–12 (**Figure 6B**) as compared with mice treated with the control or mock CAR-T cells. Likewise, mice treated with XCR1 CAR-T cells had a delayed initial EAE onset (**Figure 6C**). Following the initial delay in EAE onset, mice treated with XCR1 CAR-T cells exhibited similar kinetics of EAE score, weight loss, and 100% disease incidents which matched that of control and mock CAR-T cell-treated mice from days 13 to 24 (**Figures 6A–C**), suggesting that DC1 are essential only during the early phase of passive EAE.

On day 24, the ILN, spleen, and total CNS were collected, processed, and analyzed *via* flow cytometric analysis for donor CAR-T cells and recipient (CD45.2^+^) DC subsets. XCR1 CAR-T cells successfully engrafted in the lymphoid organs and infiltrated the CNS (**Figures 6D, E**). XCR1 CAR-T cell transfer did not significantly alter the total DC frequencies (MHCII^+^ CD11c^+^) as compared with both control and mock CAR-T cell-treated mice (**Figures 6D, F**) but specifically depleted DC1 (XCR1^+^ CD11b^−^) in spleen, ILN, and CNS (**Figures 6D, G**). Overall, these results suggest that depletion of DC1 with CD8^+^ XCR1 CAR-T cells suppresses the onset of passive EAE.

### 3.7 XCR1 CAR-T cells suppress the onset of CD3^+^ T cell-induced EAE

**Figure 7:**
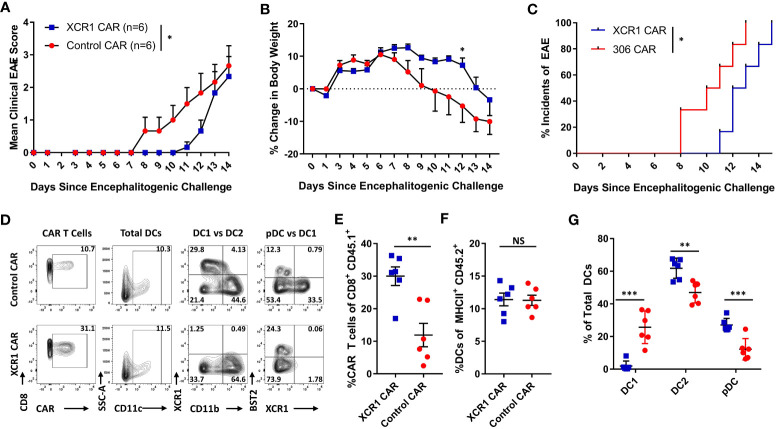
XCR1 CAR-T cells suppress the onset of CD3^+^ T cell-induced EAE. RAG2^−/−^ mice were iv injected with 1 x 10^6^ purified CD3^+^ encephalitogenic T cells, to induce EAE, on day 0. Subsequently, RAG2^−/−^ mice were injected IP with 100 ng PTx on days 0 and 2. In addition, mice were iv treated with 1 x 10^6^ XCR1 or control CD8^+^ CAR T cells (n = 6/group) on day 0. Shown **(A)** are the daily mean clinical EAE scores and **(B)** the mean percent change in body weight and **(C)** the initial day of EAE onset from day 0 through day 14 for XCR1 and control CAR-T cell-treated mice. The CNS was harvest on day 19 and analyzed *via* flow cytometry for the presence of CAR-T cells and different DC subsets. Shown **(D)** are representational dot plots; first column, CAR-T cells (CD45.1^+^ CD8^+^) assessed for CD8 (y-axis) and mCherry (x-axis); second column, total DCs (MHCII^+^) assessed for SSC-A (y-axis) and CD11c (x-axis); third column, DCs (MHCII^+^ CD11c^+^) assessed for XCR1 (y-axis) and CD11b (X-axis); fourth column, DCs assessed for BST2 (y-axis) and CD11b (X-axis). The percent CAR^+^ T cells of CD45.1^+^ donor cells **(E)** and the percent DCs of total CD45.2^+^ F4/80^−^ MHCII^+^ cells **(F)** for CAR-treated mice. Shown **(G)** are the percent DC1, DC2, and pDC, of total DCs. Statistical significance was analyzed by use of **(A)** Wilcoxon matched pairs test, **(B)** two-way repeated measures ANOVA, **(C)** Gehan–Breslow–Wilcoxon test and **(D-G)** unpaired two-tailed t test. *p < 0.05, **p < 0.01, ***p < 0.001. Error bars represent SEM.

The role of CD8^+^ T cells in EAE is poorly understood, and CD8^+^ T cells can likely have dual roles as inflammatory or suppressive mediators under different contexts. Because XCR1^+^ DC1 are cross-presenting DC and critical for CD8^+^ T-cell responses, we tested if DC1 depletion suppressed EAE induced with pooled effector CD4^+^ and CD8^+^ T cells. RAG2^−/−^ mice (n = 6/group) were iv injected with 1 x 10^6^ purified CD3^+^ encephalitogenic T cells from MOG^35–55^)/CFA-immunized WT donor mice, alongside 1 x 10^6^ XCR1, control, or mock-transduced CD45.1^+^ CD8^+^ T cells. As seen with CD4^+^ T cell-induced passive EAE (**Figure 6**), XCR1 CAR-T cells suppressed the onset of CD3^+^ T cell-induced EAE (**Figures 7A–C**). Specifically, XCR1 CAR-T cells significantly suppressed the mean clinical EAE score (**Figure 7A**) and significantly reduced weight loss on day 12 (**Figure 7B**) compared with mice treated with control CAR-T cells. Likewise, there was a significant delay in the EAE onset in XCR1 compared with control CAR-T cell-treated mice (**Figure 7C**). XCR1 CAR-T cells engrafted in the CNS (**Figures 7D, E**) and did not affect the total DC frequencies (**Figure 7F**) but ablated DC1, increased DC2, and increased pDC (**Figure 7G**) in the CNS compared with control CAR-T cell-treated mice. Collectively, these data suggest that DC1 depletion suppresses the onset of CD3^+^ T cell-induced passive EAE.

### 3.8 Diphtheria toxin-mediated DC1 depletion suppressed the onset of passive CD4^+^ T-cell adoptive transfer EAE but not active EAE

**Figure 8:**
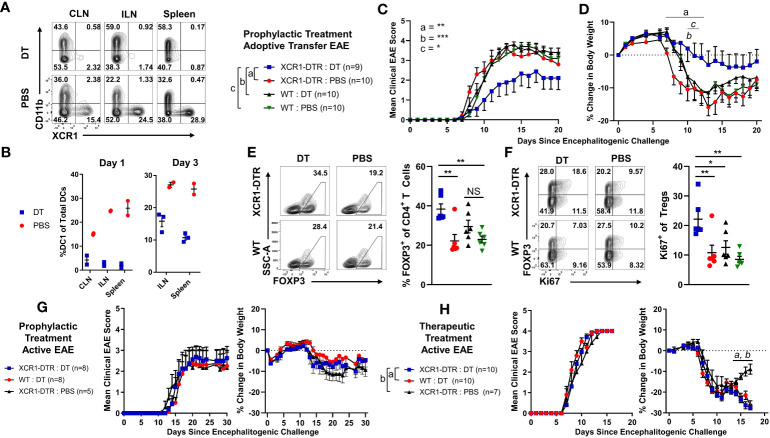
Diphtheria toxin (DT)-mediated DC1 depletion suppressed the onset of passive CD4^+^ T cell adoptive transfer EAE but not active EAE. **(A, B)** XCR1-DTR mice (n = 2–3/group) were IP injected with 400 ng DT or PBS on day 0. The CLN, ILN, and spleen were harvest 1 or 3 days later and analyzed *via* flow cytometry for DC1. Shown **(A)** are representational dot plots of DCs (MHCII^+^ CD11c^+^) from each lymphoid organ assessed for CD11b (y-axis) and XCR1 (x-axis) from mice treated with DT (top) or PBS (bottom). Graphed are the percent DC1 (XCR1^+^ CD11b^−^) of total DCs (MHCII^+^ CD11c^+^) in the ILN, CLN, and spleen for day 1 (left) and day 3 (right). **(C–F)** XCR1-DTR or WT mice (n = 9–10/group) were iv injected with 2 × 10^6^ purified CD4^+^ encephalitogenic T cells on day 0. Subsequently, mice were injected IP with 120 ng PTx on days 0 and 2 and with 200 ng DT or PBS IP every other day from days 0 to 15. Shown **(C)** are the daily mean clinical EAE scores and **(D)** the percent change in body weight from day 0 through day 20. The CNS was harvested on day 20 from six representational mice from each group and analyzed *via* flow cytometry for the presence Tregs. Shown **(E)** are representational dot plots of CD4^+^ T cells (TCRβ^+^ CD4^+^) assessed for SSC-A (y-axis) and FOXP3 (x-axis) and graphed percent Tregs of total CD4 T cells for each group. Shown **(F)** are CD4^+^ T Cells (TCRβ^+^, CD4^+^) assessed for FOXP3 (y-axis) and Ki67 (x-axis), and graphed are the percent Ki67^+^ FOXP3^+^ Treg of total CD4^+^ T cells for each group. **(G, H)** On day 0, XCR1-DTR or WT mice (n = 7–10/group) were injected with MOG^35–55^ in CFA followed by 120 ng PTx IP on days 0 and 2. **(G)** Mice were treated with 400 ng of DT IP every other day 0 to 14. **(H)** Mice were treated with 400 ng DT every other day from days 10 to 16. Shown **(G, H)** are the mean clinical EAE scores and the percent change in body weight over days since encephalitogenic challenge for each group. Statistical significance was analyzed by use of **(C)** Friedman test followed by Dunn’s multiple comparisons test, **(E, F)** one-way ANOVA Tukey’s multiple comparisons test, and **(D, H)** two-way repeated measures ANOVA, *p < 0.05, **p < 0.01, ***p < 0.001. Error bars represent SEM. Data are representative of two independent experiments.

We next wanted to determine if CD8^+^ XCR1 CAR-T cell treatment suppressed the onset of EAE *via* DC1 depletion, or through other possible mechanisms (**Figure 8**). For example, CD8^+^ CAR-T cells might directly compete with encephalitogenic CD4^+^ T cells for growth factors or CD8^+^ CAR-T cells could produce large amounts of cytokines such as IFN-γ which can suppress EAE (35). Therefore, we tested an alternative mode of DC1 depletion and utilized XCR1-DTR mice, in which DT administration leads to selective DC1 depletion. To test DT-mediated DC1 depletion, we IP-injected XCR1-DTR mice (n = 2–3/group) with 400 ng of DT or PBS and measured DC1 percentages in lymphoid organs *via* flow cytometric analysis 1 or 3 days later (**Figures 8A, B**). DT administration completely ablated DC1 (XCR1^+^ CD11b^−^) in that less than 0.5% of total DC were DC1 in the CLN, ILN, and spleen of mice treated with DT compared with ~13%–22% DC1 in PBS-treated mice (**Figure 8B**). On day 3, DC1 had started to recover in that ~10%–15% of DC were DC1 in the ILN and spleen of DT-treated mice compared with 25%–27% DC1 in PBS-treated mice (**Figure 8B**). A dose of 200-ng DT was also sufficient to deplete XCR1-expressing DC (data not shown). Therefore, we selected to administer either 200 or 400ng DT every other day to maintain sufficient DC1 depletion that would mimic XCR1 CAR-T cell-mediated DC1 depletion.

To test the effects of DC1 depletion on passive EAE, XCR1-DTR, or WT mice were iv injected with CD4^+^ encephalitogenic T cells on day 0 alongside 120 ng PTx, IP on days 0 and 2. PBS or 200 ng DT was administered every other day from day 0 to day 14. As seen with CAR-T cell-mediated depletion of XCR1^+^ cells (**Figures 6**, **7**), DT-mediated DC1 depletion suppressed the onset of passive EAE (**Figures 8C, D**). Specifically, DT-treated XCR1-DTR mice (DC1 depleted) had significantly reduced mean clinical EAE scores compared with DC1-sufficient, PBS-treated XCR1-DTR mice, and PBS and DT-treated WT mice (**Figure 8C**). In addition, DT-treated XCR1-DTR mice exhibited significantly less weight loss on day 11 compared with PBS-treated XCR1-DTR mice and PBS and DT-treated WT mice (**Figure 8D**). At the end of the experiment on day 20, the CNS was harvested and analyzed *via* flow cytometric analysis for T cell subsets. There was no difference between the frequencies of MOG^35–55^ tetramer^+^ T cells in the CNS between groups (data not shown). However, there was a significant increase in polyclonal Tregs in DT-treated XCR1-DTR mice compared with PBS-treated XCR1-DTR and WT mice (**Figure 8E**). In addition, Tregs from XCR1-DTR mice treated with DT had increased Ki67, suggesting increased proliferation of Tregs (**Figure 8F**).

We repeated these findings with CD3^+^ and CD4^+^ T cell-induced passive EAE. XCR1-DTR mice were iv injected with purified CD3^+^ or CD4^+^ encephalitogenic T cells on day 0 alongside 120 ng PTx IP on days 0 and 2. PBS or 400 ng DT was administered every other day from day 0 to day 14 (**Supplemental Figures 9A–F**). DT-treated XCR1-DTR mice with either CD4^+^ and CD3^+^ T cell-induced passive EAE had significantly reduced mean clinical EAE scores compared with PBS-treated mice (**Supplemental Figures 9A, B**). At the end of the experiment on day 21, the total CNS was harvested and analyzed *via* flow cytometric analysis for T cell subsets. There was no difference between the percent of tetramer^+^ MOG^35–55^-specific T cells of total CD4^+^ T cells in the CNS of DT or PBS-treated mice in either CD4^+^ or CD3^+^ T cell-induced passive EAE (**Supplemental Figure 9D**). However, there was a significant increase in Tregs among the polyclonal and tetramer^+^ MOG^35–55^-specific T cells in both CD4^+^ or CD3^+^ T cell-induced passive EAE (**Supplemental Figures 9C, E, F**). Together, these data suggest that DC1 depletion is sufficient to suppress the onset of passive EAE and increase the Treg pool among MOG^35–55^ effector CD4^+^ T cells and polyclonal T cells.

To test the effects of DC1 depletion on active EAE, XCR1-DTR, or WT mice were immunized with MOG^35–55^/CFA subcutaneously on day 0 alongside 120 ng PTx IP on days 0 and 2 (**Figures 8G, H**). Mice were IP injected with PBS or 400 ng DT every other day either prophylactically on days 0 through 14 (**Figure 8G**) or therapeutically on days 10 through 16 (**Figure 8H**). Interestingly, prophylactic DC1 depletion had no detectable effect on the mean clinical EAE score or the initial weight (**Figure 8G**). Likewise, therapeutic DC1 depletion had no detectable effects on the EAE scores (**Figure 8H**) during active EAE. However, therapeutic DT treatment of both XCR1-DTR and WT mice led to significantly increased weight loss compared with PBS-treated XCR1-DTR mice. These findings were consistent with a previous report in which DT administration was toxic in immunized (CFA + PTx) WT mice (43). Therefore, we concluded that DC1 depletion was ineffective during the onset of active EAE, and experimental constraints prevent thorough testing of therapeutic DC1 depletion in active and passive EAE.

### 3.9 XCR1 and MOG CAR-Tregs are functional and suppresses EAE

**Figure 9:**
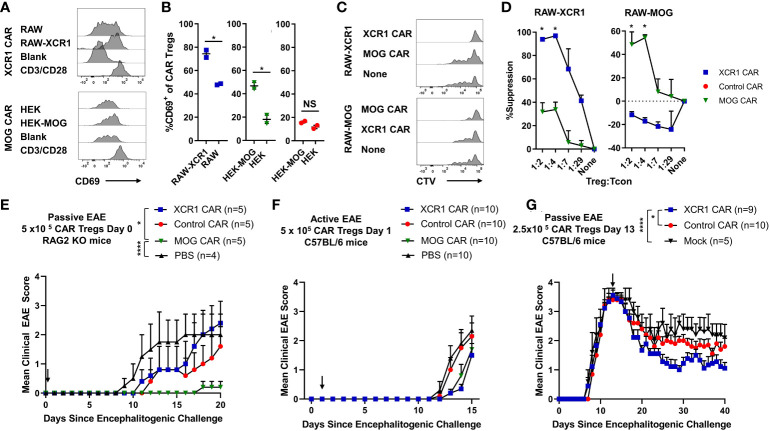
XCR1 and MOG CAR-Tregs are functional and suppress EAE. **(A, B)** 50,000 XCR1, control, or MOG CD4^+^ CAR-Treg were cocultured with 5 x 10^5^ irradiated RAW, RAW–XCR1, HEK, or HEK–MOG overnight in complete media with 3 ng/mL IL-2. Shown **(A)** are representational histograms of CD69 (x-axis) expression of XCR1 (top) or MOG (bottom) CAR-Treg cocultured with respective cell lines, mouse T-activator beads “CD3/CD28” at a 10:1 ratio, or alone. Graphed **(B)** are the percent CD69^+^ of XCR1 (left), MOG (middle), or control (right) CAR-Treg following cocultured with respective cell lines. **(C, D)** 25,000 CTV-stained OTII responder T cells were cocultured with 50,000 irradiated (30 Gy) BMDC and 50,000 RAW, RAW–XCR1, or RAW–MOG cells in complete media with 64 nM OVA323-339. XCR1 or MOG CAR-Treg were titrated and added at designated ratios. Shown **(C)** are representative histograms of CTV dilution of responder OTII T cells under each condition. Graphed **(D)** is the percent suppression of responder OTII T cells at designated ratios with XCR1 or MOG CAR-Tregs cocultured with RAW–XCR1 (left) and RAW–MOG (right). **(E)** RAG2^−/−^ (n = 4–5/group) were iv injected with 1 x 10^6^ purified CD4^+^ encephalitogenic T cells and 5 × 10^5^ XCR1, control, MOG, or mock CAR-Treg on day 0 and IP with 120 ng PTx on days 0 and 2. Shown **(E)** are the daily mean clinical EAE scores from day 0 through day 20. **(F)** C57BL/6 mice (10/group) were injected with MOG^35–55^ in CFA followed by 120 ng PTx IP on days 0 and 2. 5 × 10^5^ XCR1, control, MOG CAR-Treg, or PBS were injected on day 1. Shown **(F)** are the mean clinical EAE scores from day 0 through day 15. **(G)** C57BL/6 mice (5–10/group) were injected with 2 × 10^6^ purified CD4^+^ encephalitogenic T cells followed by 120 ng PTx IP on days 0 and 2. Then, 2.5 × 10^5^ XCR1, control, or mock CAR-Treg were iv injected on day 13. Shown **(G)** are the mean clinical EAE scores from day 0 through day 40. Statistical significance was analyzed by use of **(B, D)** unpaired two-tailed t test and **(E, G)** Friedman test followed by Dunn’s multiple comparisons test for day 8 through end of experiment. *p < 0.05, ****p < 0.0001 Error bars represent SEM. Data **(A-D)** are representative of three independent experiments.

Polyclonal and antigen-specific Tregs can suppress EAE, with CNS-specific Tregs having greater efficacy. In addition, Tregs can modulate the function of DCs as a mechanism of immune suppression. However, it is unclear if DC-directed CAR-Tregs can suppress inflammation. Thus, we sought to determine if XCR1 CAR-Tregs directed to DC1 were suppressive and could inhibit EAE (**Figure 9**). To test the *in vitro* function of XCR1 CAR-Tregs, XCR1, control, or mock CAR-Tregs were cocultured with irradiated RAW, HEK, or cell lines stably transduced with XCR1 (RAW–XCR1 and HEK–MOG) overnight (**Figures 9A, B**). Next, the CAR-Tregs were assessed for CD69 expression, as a marker of cell activation, *via* flow cytometric analysis. XCR1 CAR-Tregs specifically upregulated CD69 expression, in that ~75% expressed CD69 in response to RAW–XCR1 compared with ~45% when cultured with RAW cells (**Figures 9A, B**). Likewise, MOG CAR-Treg specifically upregulated CD69 expression, in that ~45% expressed CD69 in response to HEK–MOG compared with ~18% when cultured with HEK cells. Control CAR-T had similar levels of CD69 expression (~18%) when cocultured with either HEK–MOG or HEK cells (**Figures 9A, B**). Therefore, both XCR1 and MOG CAR-Tregs exhibited CAR-specific activation and had increased CD69 expression in response to cell lines expressing either XCR1 or MOG, respectively. To test the suppressive function of XCR1 and MOG CAR-Tregs, CTV-stained OTII responder T cells were cocultured with irradiated BMDC and RAW, RAW–XCR1, or RAW–MOG cells with the OTII peptide (OVA323-339). XCR1 or MOG CAR-Tregs were titrated and added at decreasing ratios from 1:1 to 1:29, Treg to effector OTII T cells. Pure DC2 BMDC, acted as APC to present OTII peptide to OTII T cells, and RAW cell lines expressing either XCR1 or MOG were used to activate XCR1 or MOG CAR-Tregs, respectively. The XCR1 CAR-Tregs were more efficient in suppressing OTII proliferation resulting in ~3-fold increased suppression over that of MOG CAR-Tregs when cocultured with RAW–XCR1 cells (**Figures 9C, D**). Similarly, MOG CAR-Tregs were more efficient in suppressing OTII proliferation resulting in ~4.5-fold more suppression over that of XCR1 CAR-Tregs when cocultured with RAW–MOG cells.

Next, we characterize the cytokine expression profiles of XCR1 CAR-Tregs cells in coculture with DC, which can also produce cytokines in response to Tregs, *in vitro*. XCR1, control or mock, CD4^+^, CD8^+^ CAR-T cells, and CD4^+^ CAR-Tregs were cocultured with BMDCs for 48 h and then the supernatant was collected and cytokine production was analyzed *via* MSD assay (**Supplemental Figures 3A–O**). XCR1 CAR-Tregs produced high amounts of IL-10 compared with mock and control CAR-T cells and compared with CD4^+^ and CD8^+^ XCR1 CAR-T cells (**Supplemental Figure 3G**). Interestingly, XCR1 CAR-Tregs also made increased amounts of MIP-2 and MCP-1 compared with CD4^+^ and CD8^+^ CAR-T cells (**Supplemental Figures 3K, N**). In addition, XCR1 CAR-Treg had an increased cytokine profile (IL-5, IL-4, IL-6, TNF-α, IL-2, and IL-12) in comparison with control or mock CAR-Tregs; however, the cytokine production was significantly less that that produced by CD4^+^ XCR1 CAR-T cells. In all, we show that XCR1 CAR-Tregs are activated by BMDC and produce large amounts of IL-10 and have a decreased cytokine profile as compared with CD4^+^ XCR1 CAR-T cells.

To determine if XCR1 CAR-Tregs suppressed or depleted DC1 *in vivo*, we adoptively transferred 3 × 10^6^ XCR1, control, or mock CAR-Tregs into RAG2^−/−^ mice. On day 5, the spleen and ILN were analyzed for DC subsets and activation markers (**Supplemental Figures 10A–E**). There was no decrease in total DC, DC1, DC2, or pDC subsets (**Supplemental Figures 10B, C**), and there was no discernible decrease in the percent of CD40^+^, CD80^+^, or CD86^+^ DC1 cells (**Supplemental Figures 10D, E**).

To determine if XCR1 and MOG CAR-Treg were suppressive *in vivo*, RAG2^−/−^ mice (4–5/group) were injected with 1 x 10^6^ encephalitogenic CD4^+^ T cell and 5 × 10^5^ XCR1, MOG, control CAR-Tregs, or PBS on day 0. MOG CAR-Tregs almost completely prevented EAE, compared with PBS and control CAR-Treg-treated mice (**Figure 9E**). However, XCR1 CAR-Treg-treated mice had a slight non-significant delay in the onset of passive EAE compared with PBS-treated mice. Therefore, MOG and not XCR1 CAR-Treg were most efficacious in preventing passive EAE in RAG2^−/−^ mice.

Next, we sought to determine if XCR1 CAR-Tregs were effective in active EAE in immunocompetent mice. C57BL/6 mice (5–10/group) were injected with MOG^35–55^ in CFA followed by 120 ng PTx IP on day 0. Subsequently, mice were iv injected prophylactically on day 2 with 5 × 10^5^ XCR1, control, or MOG CAR-Tregs (**Figure 9F**). Prophylactic treatment with either MOG or XCR1 CAR-Tregs mildly suppressed EAE onset and led a non-significant decrease in the mean EAE score from days 13 through 14 compared with PBS and control CAR-Treg-treated mice (**Figure 9F**). Next, we tested if XCR1-CAR Tregs could suppress passive EAE after adoptive transfer in a therapeutic setting. Mice were iv injected with CD4^+^ encephalitogenic T cells on day 0 alongside 120 ng PTx IP on days 0 and 2. Then, on day 13, mice were treated with 2.5 × 10^5^ XCR1, control, or mock CAR-Treg (**Figure 9G**). Therapeutic XCR1 CAR-Treg treatment suppressed passive EAE and led to a significant decrease in the EAE scores compared with both mock and control CAR-Tregs (**Figure 9G**). Therefore, we concluded that XCR1 CAR-Tregs can suppress passive EAE in immunocompetent C57BL/6 mice.

## 4 Discussion

To our knowledge, this is the first reported therapeutic strategy to specifically deplete DC1 *in vivo* in mice. Likewise, these data represent the first DC-targeted CAR-T cell and CAR-Treg therapies. DC depletion is gaining traction as a therapeutic intervention for autoimmunity because antibody-mediated depletion of pDC showed efficacy in animal models of SLE and is currently in clinical trials (44–46). These promising results suggest that DC-targeted therapies may provide a therapeutic modality to relieve and treat autoimmunity. CAR-T cells can have several advantages over antibodies as therapeutics because T cells are long lived, self-renew, migrate to target tissues, and exhibit increased antigen specific cytotoxicity, whereas antibodies are constrained by pharmacological half-life and must be chronically administered. Together, these attributes make CAR-T cell therapy attractive. 

CAR-T cells have been extensively evaluated in preclinical animal models of cancer; however, applications of CAR-T cells and CAR-Tregs in autoimmunity and metabolic disease are still being explored. CAR-T cells or CAR-Tregs can potentially deplete or suppress pathogenic mediators across a variety of autoimmune diseases. For example, CAR-Tregs have shown efficacy in a variety of preclinical animal models of autoimmune disease including EAE, colitis, transplant rejection, allergic disease, rheumatoid arthritis, and antidrug responses (47). Likewise, CAR-T cells have shown efficacy in preclinical animal models of autoimmune disease including EAE, GvHD, SLE, pemphigus vulgaris, and T1D (47).

Here, we show that DC-targeted CD8^+^ CAR-T cells were able to suppress EAE in lymphopenic host (**Figures 6**, **7**). However, XCR1 CAR-T cells failed to engraft or deplete DC1 in immunocompetent mice (**Figure 3**). These findings are consistent with both preclinical and clinical findings that CAR-T cells often require lymphodepletion for both engraftment and cytotoxic activity (48, 49). Interestingly, control (anti-human-myostatin) CAR-T cells failed to durably engraft in immunocompromised RAG2^−/−^ mice, most likely due to the lack of endogenous antigen expression that would have allowed stimulation of the CAR leading to proliferation and persistence of the CAR-T cells.

IL-2 is a critical cytokine required for T-cell viability and has been shown to increase CAR-T cell activity in other studies (50). Therefore, we initially tested IL-2 in combination with CAR-T cell transfer. However, we found that IL-2 was not required for XCR1 CAR-T cell activity in the context of lymphopenic RAG2^−/−^ host (**Figure 3**). Likewise, we did not note any notable differences in experiments in which we used or excluded IL-2.

Although promising, there are numerous barriers to implementing CAR-T cell or CAR-Treg-based therapies, including high cost of manufacturing, possible off cytotoxicity, and cytokine release syndrome when using CAR-T cells and possible CAR Treg instability. Here, we show that CD4^+^ CAR-T cells produce higher amounts of proinflammatory cytokines (**Figure 2**) compared with CD8^+^ CAR-T. Therefore, CD8^+^ CAR-T cells may represent a safer modality for cell depletion. Caution should be taken when advancing strategies that deplete DC1, as DC1 are protective in intestinal inflammation (51), DC1 are implicated in the clearance of cancers (10), and DC1 play a critical role in viral and bacterial clearance. Also, CAR targets should be thoroughly vetted to ensure that receptor expression is limited to target tissue alone. Based on Tabula muris, XCR1 expression is predominantly seen in myeloid cells, but some expression is also observed in non-immune cells such as a subset of fibroblasts (in the heart) and epithelial cells (in the kidney). Therefore, before progressing a XCR1 CAR-T therapy, off-target toxicity should be investigated. However, we did not detect overt signs of autoimmunity or toxicity in our studies.

DC1-targeted XCR1 CAR-Treg showed modest efficacy in a therapeutic setting in passive Th1-driven EAE (**Figure 9G**). Because DC1 are found systemically, in both lymphoid and non-lymphoid tissues, it is likely that XCR1 CAR-Tregs are systemically and constitutively activated. Therefore, XCR1 CAR-Tregs-mediated suppression might be due to the introduction of systemically activated immunosuppressive Tregs through both contact-dependent and independent manners. Alternatively, increases in DC populations in the CNS during EAE could result in increased XCR1 CAR-Tregs in the CNS and suppression of EAE. XCR1 CAR-Treg could also directly augment DC activity to resolve EAE; however, the exact mechanism is unknown. We also compared XCR1 CAR-Tregs with MOG CAR-Treg, which targeted the CNS, in a passive model of EAE (**Figure 9E**). MOG CAR-Tregs were profoundly more efficient at blocking the development of passive EAE as compared with XCR1 CAR-Tregs (**Figure 9E**). Thus, CNS-specific MOG CAR-Tregs exhibited enhanced protection against CNS-directed autoimmunity compared with DC1-specific XCR1 CAR-Tregs. This is consistent with previous reports that MOG CAR-Tregs migrate to CNS, are activated, and suppress EAE (52, 53).

DT-mediated depletion of DC1 in XCR1-DTR mice suppressed the development of passive EAE (**Figures 8A–D**). Interestingly DC1 depletion had no effect on active EAE, in which mice are immunized with MOG/CFA, but suppressed passive EAE, in which T cells are adoptively transferred (**Figure 8**). These data are consistent with previous reports in which total DC depletion exacerbated active EAE but suppressed passive EAE (8, 9, 17, 25–28). Taken together, these results suggest that DC1 play a critical role in presenting endogenous autoantigens to activate pathogenic T cells under homeostatic conditions where there is a lack of tissue damage and proinflammatory cues. However, DC1 may play a more passive role or even regulatory role in adjuvant-primed environments where there are proinflammatory cues and exogenous autoantigen, leading to antigen presentation by activated non-DC antigen-presenting cells such as macrophages/B-cells. These results collectively suggest that under homeostatic conditions, DC1 are a major contributor to inflammation and serve to activate pathogenic T cells. Importantly, our findings provide more precise insight into the proinflammatory role of DC1 in autoimmunity, compared with previous studies that relied on other DC1 depletion methods (29–31). Using XCR1-DTR mice, we were able to precisely target and deplete only DC1, because XCR1 expression is limited to DC1 alone. Furthermore, our research also sheds light on the previously underappreciated involvement of DC1 in the activation of autoreactive CD4^+^ T cells in EAE.

DC1 depletion increased polyclonal Tregs and Treg proliferation in EAE mice (**Figure 8**). Possible but not exclusive explanations include the absence of DC1 or the skewing of endogenous antigen presentation to DC2 and pDC favoring Treg expansion. DC1 are a significant source of IL-12, which suppresses Treg induction, proliferation, and FOXP3 expression levels (54). Likewise, pDC and DC2 have been shown to expand Tregs and increase Treg suppressive function (55, 56). Collectively, our results here provide evidence that the absence of DC1 and increase of pDC and DC2 lead to increased polyclonal Tregs during active autoimmunity.

Overall, we provide proof of concept that CAR-T can selectively, systemically, and durably deplete DC1 to modulate autoimmunity and DC1-targeted CAR-Tregs can suppress passive Th1-induced EAE. In addition, these data suggest that DC1 play a pathogenic role in passive EAE and that DC1 depletion and/or expansion of pDC and DC2 populations favors Treg expansion.

## Data availability statement

The raw data supporting the conclusions of this article will be made available by the authors, without undue reservation.

## Ethics statement

The animal studies were approved by The Department of Comparative Medicine at Amgen South San Francisco and the Charles River Accelerator and Development Lab. The studies were conducted in accordance with the local legislation and institutional requirements. Written informed consent was obtained from the owners for the participation of their animals in this study.

## Author contributions

CM designed and performed experiments; analyzed and interpreted data; made the figures; and wrote the manuscript with AC. CB designed and described the XCR1-DTR mice. SY, AS, and AR performed experiments. AC and HP designed experiments and interpreted data.
