# Supplementary material for: CAR-T cells and CAR-Tregs targeting conventional type-1 dendritic cell suppress experimental autoimmune encephalomyelitis
Source: Front Immunol. 2023 Oct 27;14:1235222. doi: 10.3389/fimmu.2023.1235222 (PMC10641730; doi:10.3389/fimmu.2023.1235222)
Supplement: Supplementary file 1 [file DataSheet_1.docx]

**Supplemental Figure 1:**


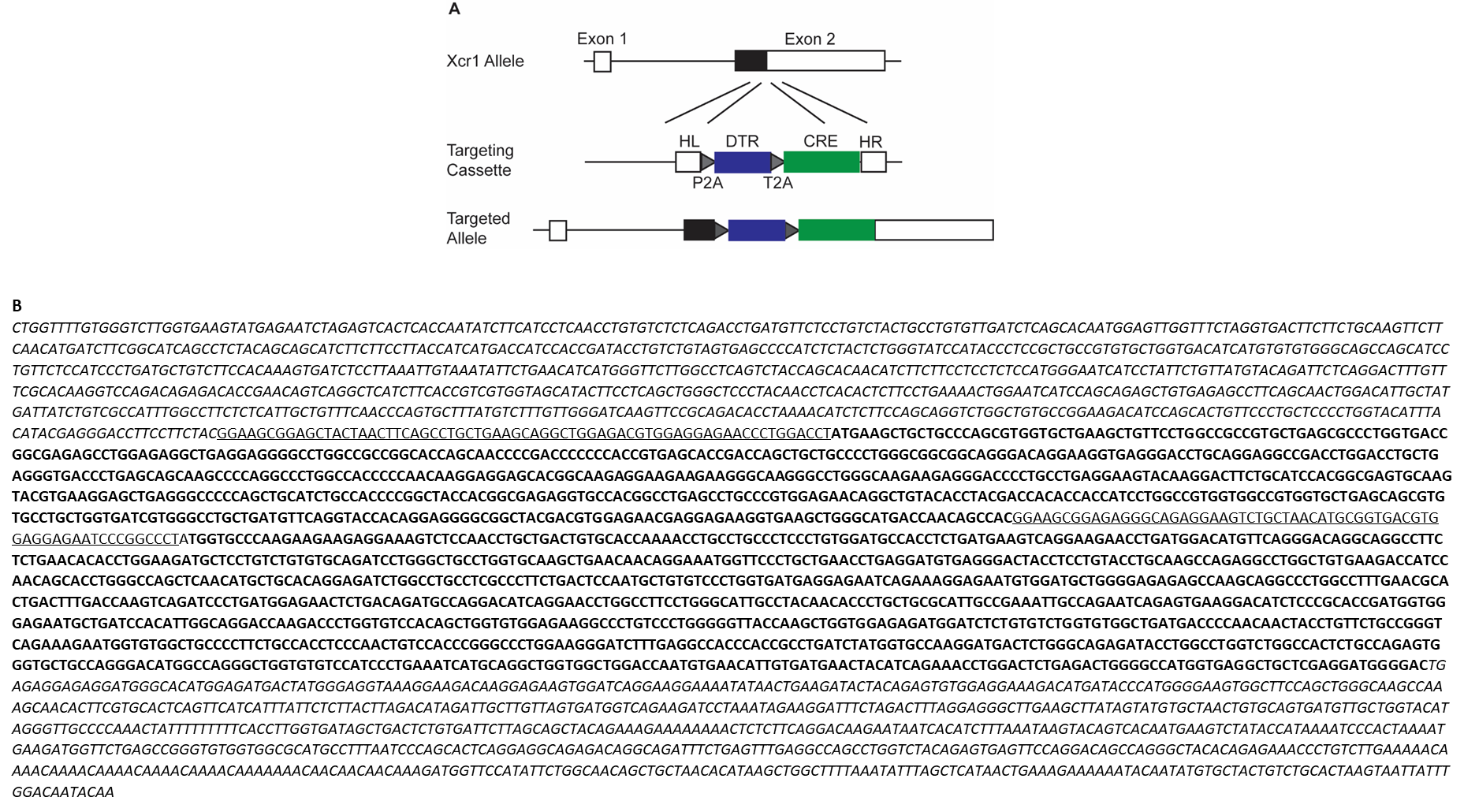


**Supplemental Figure 1: Targeting Strategy for the generation of XCR1-DTR-cre mice.**

(A) The *Xcr1* WT locus, targeting cassette and targeted allele are shown (A). The *Xcr1* locus contains a non-coding first exon and a second exon encoding the entirety of the XCR1 protein. Non-coding regions are shown in white boxes, while coding regions are shaded. The targeting cassette contains sequences for the last 800 amino acids (aa) of the *Xcr1* coding region as left homology arm (HL), P2A, Diphtheria toxin receptor, T2A, Cre recombinase (CRE) and the first 799 aa of the 3’UTR as right homology arm (HR). The targeted allele is depicted with the DTR-Cre expression cassette inserted downstream of the last coding amino acid of XCR1. (B) Deoxyribonucleic acid sequence of the targeting cassette, homology arms are italicized, 2A sequences underlined, DTR and CRE sequences in bold.

**Supplemental Figure 2:**


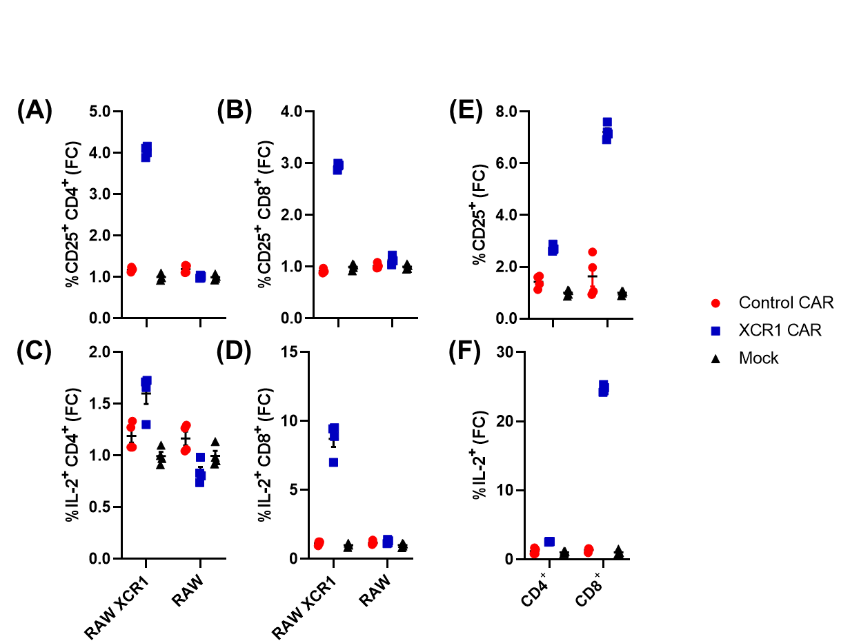


**Supplemental Figure 2: XCR1 CAR T cells were activated in response XCR1^+^ cells.**

(A-F) 1 x 10^5^ CD4^+^ or CD8^+^, XCR1, control or mock CAR T cells were cocultured with 50,000 irradiated RAW, irradiated RAW-XCR1, or BMDC for 3 days in complete media with 3ng/mL IL-2. CAR T cells were assessed for activation markers via flow cytometry. Graphed are the fold change (FC) of %CD25^+^ for CD4^+^ (A) and CD8^+^ (B) CAR T cells, and %IL-2^+^ for CD4^+^ (C) or CD8^+^ (D) CAR T cell cultures cocultured with RAW-XCR1 or RAW. Graphed are the fold change of %CD25^+^ (E) and %IL-2^+^ (F) of CD4^+^ or CD8^+^ CAR T cell cultures cocultured with BDMC. Fold change was calculated by dividing the %CAR positive population by the mean % positive population of mock CAR T cells. Error bars represent SEM. Data are representative of two independent experiments.

**Supplemental Figure 3:**


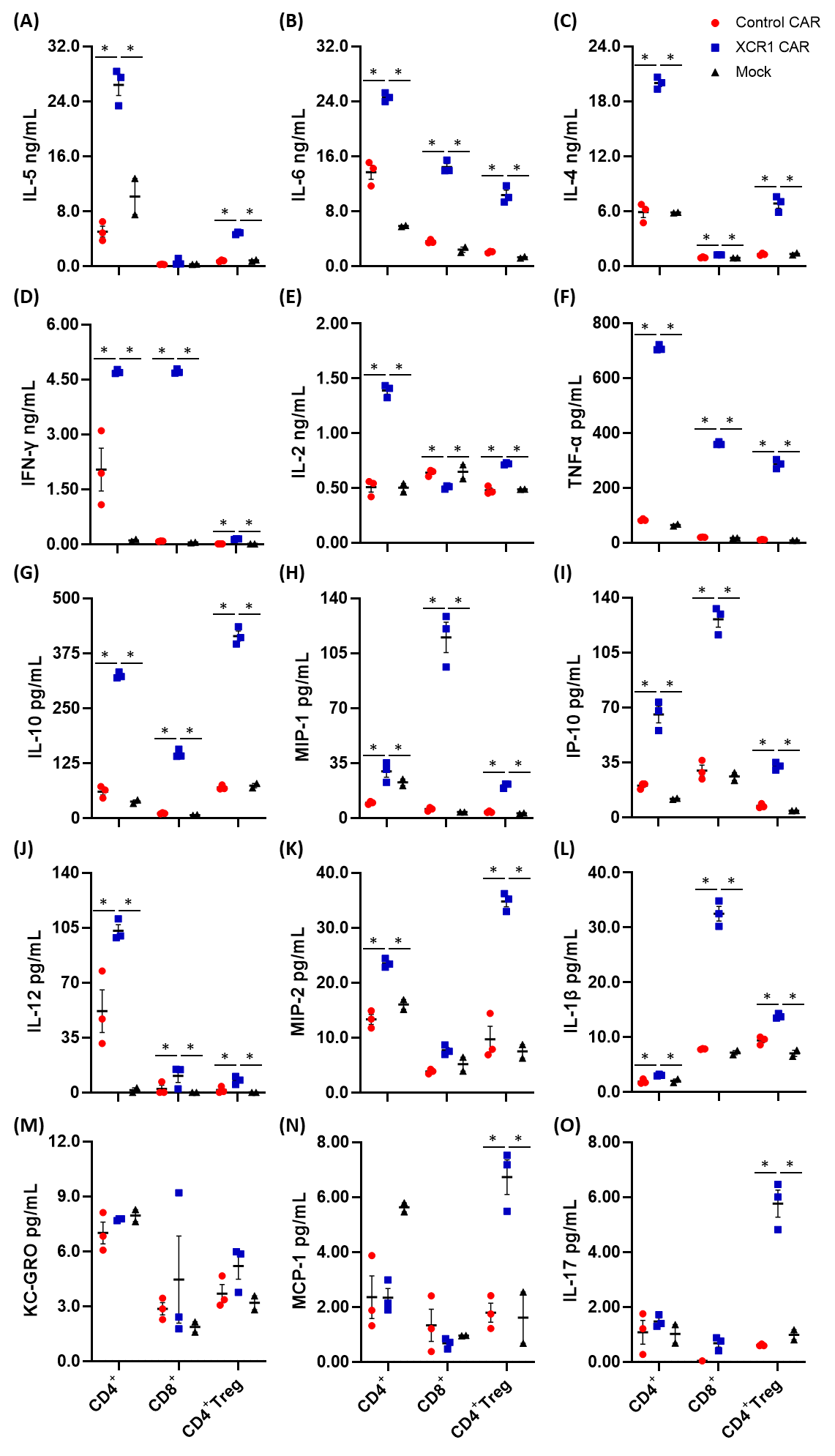


**Supplemental Figure 3: Cytokine profile of activated XCR1 CAR-T cells and CAR-Tregs.**

(A-O) 200,000 XCR1, control or mock, CD4^+^, CD8^+^, or CD4^+^ Tregs CAR-T were cocultured with 50,000 BMDC for 48 hours in T cell media with 3ng/mL IL-2. Subsequently supernatant was analyzed for indicated cytokines. Shown are graphs (A) IL-5, (B) IL-6, (C) IL-4, (D) IFN-γ, (E) IL-2, (F) TNF-α, (G) IL-10, (H) MIP-1, (I) IP-10, (J) IL-12, (K) MIP-2, (L) IL-1β, (M) KC-GRO, (N) MCP-1, and (O) IL-17 ng or pg/mL for each group. Statistical significance was analyzed by use of one-way ANOVA Tukeys multiple comparisons test *p ˂ 0.05. Error bars represent SEM.

**Supplemental Figure 4:**


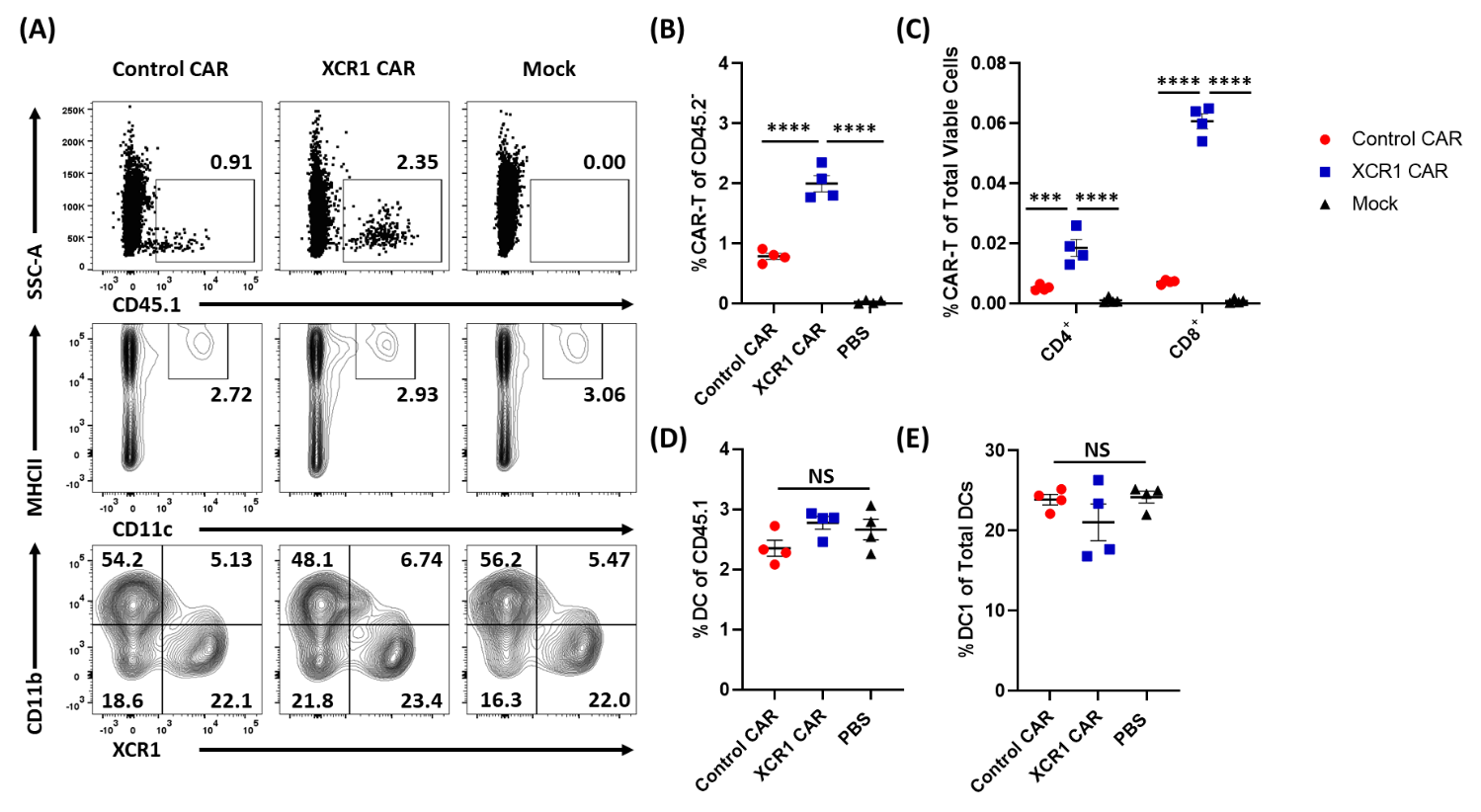


**Supplemental Figure 4: XCR1 CAR-T cells transiently persist but fail to deplete DC1 in immunocompetent mice.**

(A-E) CD45.2 mice were iv injected with PBS, 10 x 10^6^ XCR1, or control CD45.1^+^ CD3^+^ CAR-T (n=4/group). On days 8 spleens were collected from treated mice and analyzed via flow cytometry for CAR-T cells and DC subsets. Shown (A) are representative dot plots of viable CD45.2^-^ cells assessed for CD45.1 (x-axis) and SSC-A (y-axis), CD45.2^+^ cells assessed for MHCII (y-axis) and CD11c (x-axis), and DCs assessed for CD11b (y-axis) and XCR1 (x-axis). Graphed are the percent (B) CD45.1^+^ of total CD45.2^-^ cells, (C) the percent CD4^+^ or CD8^+^ CAR-T cells of total viable cells, percent DCs (MHCII^+^ CD11c^+^) of total CD45.1^+^, and percent DC1 (XCR1^+^ CD11b^-^) of total DCs. Statistical significance was analyzed by use of one-way ANOVA Tukeys multiple comparisons test. ***p ˂ 0.001, ****p ˂ 0.0001. Error bars represent SEM.

**Supplemental Figure 5:**


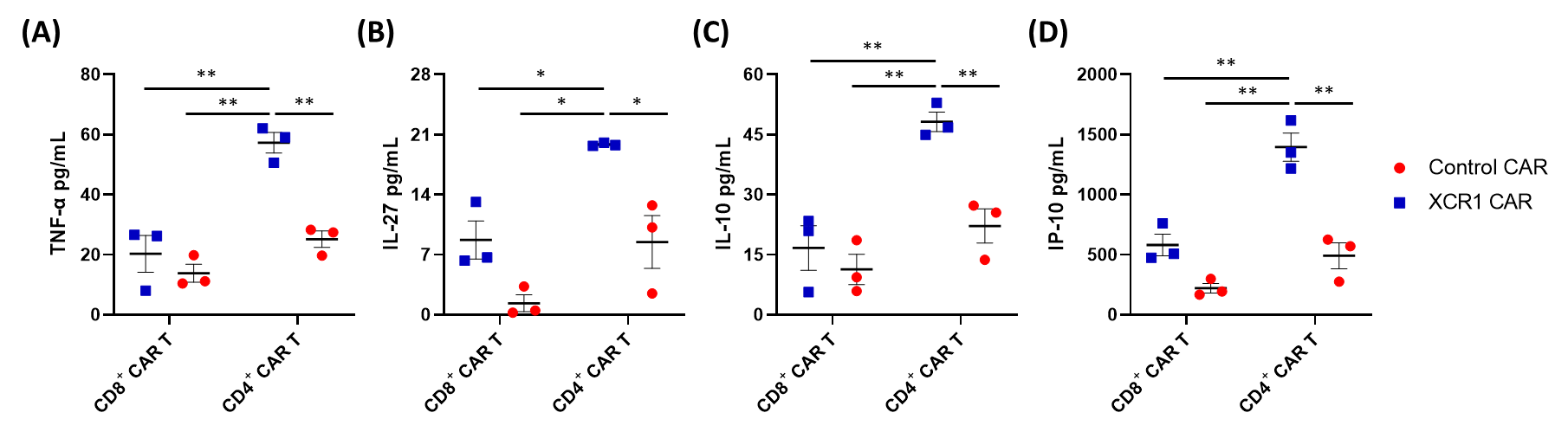


**Supplemental Figure 5: XCR1 CAR-T cell cytokine profile during engraftment in RAG2^-/-^ mice.**

RAG2^-/-^ mice were iv injected with 10^6^ XCR1, control, or mock transduced CD8^+^ or CD4^+^ CAR-T cells on day 0 (n=3/group). On day 7 serum was collected and cytokines were analyzed via MSD. Shown the concentration of (A) TNF-α, (B) IL-27, (C) IL-10, (D) IP-10 are shown for CD8^+^ and CD4^+^ CAR-T cells.


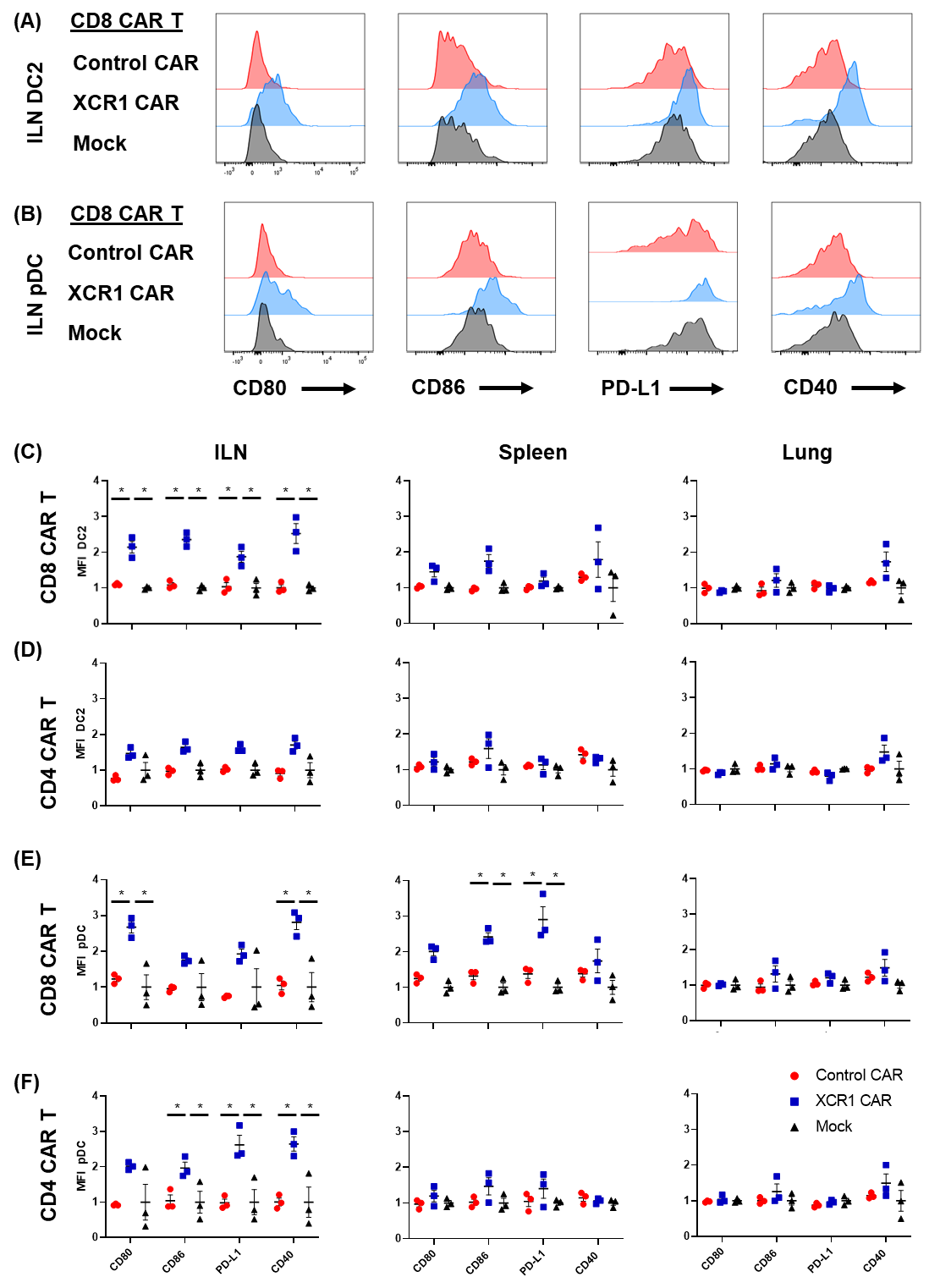
**Supplemental Figure 6:**

**Supplemental Figure 6: XCR1 CAR T cell engraftment led to DC2 and pDC activation.**

RAG2^-/-^ mice (n=3/group) were iv injected with 10^6^ XCR1, control, or mock transduced CD8^+^ or CD4^+^ CAR T cells (n=3/group) on day 0. On day 7, ILN, spleen and lung were collected and analyzed via flow cytometry for CAR T cells and DC subsets. Shown are representational histograms of recipient (A) DC2 (CD11b^+^ XCR1^-^) and (B) pDC (BST2^+^ XCR1^-^) from the ILN assessed for CD80, CD86, PD-L1 and CD40 (x-axis) of mice treated with CD8^+^ CAR T cells. Graphed are the MFI of CD80, CD86, PD-L1 and CD40 of (C, D) DC2 (CD11b^+^ XCR1^-^) and (E, F) pDC (BST2^+^ XCR1^-^) treated with (C, E) CD8^+^ CAR T or (D, F) CD4^+^ CAR T from the ILN (left) spleen (middle) and lung (right). Statistical significance was analyzed by use of one-way ANOVA Dunnett’s multiple comparisons test. Significance was shown when both control CAR and mock were significant compared to XCR1 CAR T *p ˂ 0.05, Error bars represent SEM.

**
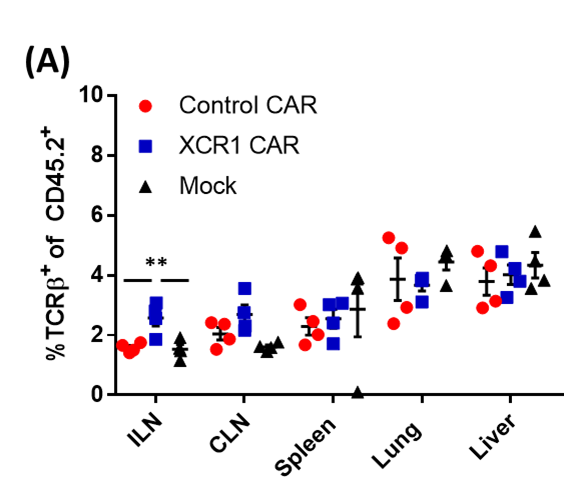
Supplemental Figure 7:**

**Supplemental Figure 7: Encephalitogenic T cell engraftment during CAR-T engraftment.**

RAG2^-/-^ mice (n=4/group) were iv injected with 10^6^ XCR1, control, or mock transduced CD45.1^+^ CD8^+^ CAR-T alongside 10^6^ encephalitogenic CD4^+^ CD45.2^+^ T cells and 10 µg of IL-2 ip on day 0. On day 6, ILN, CLN, spleen, lung and liver were collected and analyzed via flow cytometric analysis for CAR-T and DC subsets. Shown (A) the percent TCRb^+^ of CD45.1^+^ donor population for the CLN, ILN, Spleen, Lung, and Liver. Statistical significance was analyzed by use of one-way ANOVA Tukeys multiple comparisons test. **p ˂ 0.01. Error bars represent SEM.

**Supplemental Figure 8:**


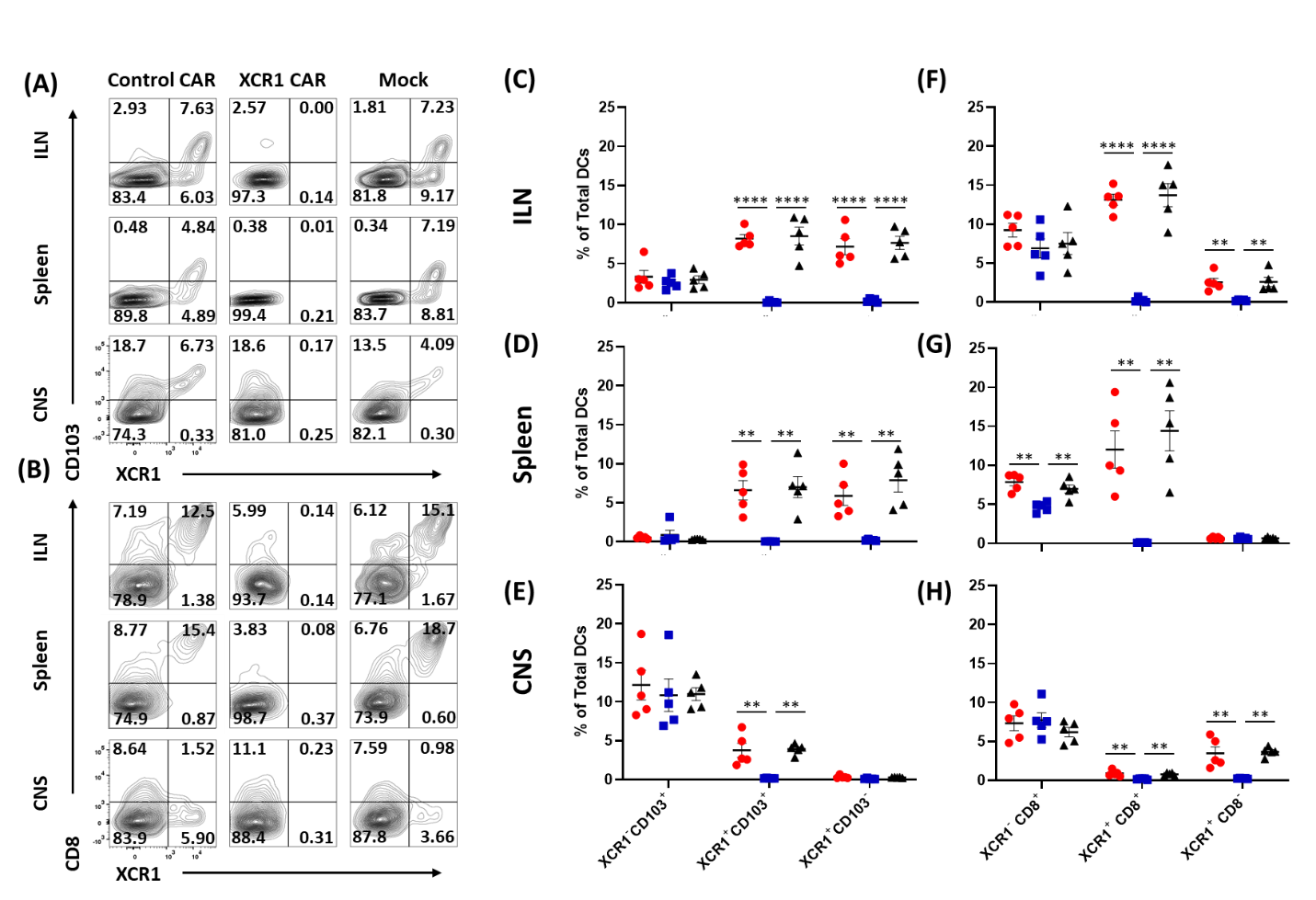


**Supplemental Figure 8: DC1 subtypes based on XCR1, CD8 and CD103 in ILN, spleen and CNS of RAG2^-/-^ mice.**

EAE was induced in RAG2^-/-^ mice by iv injection with 2 x 10^6^ CD45.2^+^ CD4^+^ encephalitogenic T cells followed by PTx on day 0 and 2. In addition, mice were treated with 2 x 10^6^ XCR1, control or mock CD45.1^+^ CD8^+^ CAR-T (n=8/group) and 10 µg of IL-2. Spleen, ILN and CNS were harvest from 5 mice from each group on day 24 and analyzed via flow cytometry for presence of DC1 subset. Shown are representative dot plots of DC (CD11c^+^ MHCII^+^) assessed for (A) CD103 (y-axis) and XCR1 (X-axis) and (B) CD8 (y-axis) and XCR1 (X-axis) from the ILN, spleen and CNS. The percent XCR1^-^ CD103^+^, XCR1^+^ CD103^+^ and XCR1^+^ CD103^-^ of total DCs (CD11c^+^ MHCII^+^) were graphed for (C) ILN, (D) spleen and (E) CNS. The percent XCR1^-^ CD8^+^, XCR1^+^ CD8^+^ and XCR1^+^ CD8^-^ of total DCs (CD11c^+^ MHCII^+^) were graphed for (F) ILN, (G) spleen and (H) CNS. Statistical significance was assessed with one-way ANOVA Tukeys multiple comparisons test. **p ˂ 0.01, ****p ˂ 0.0001. Error bars represent SEM. Data are representative of two independent experiments.

**Supplemental Figure 9:**


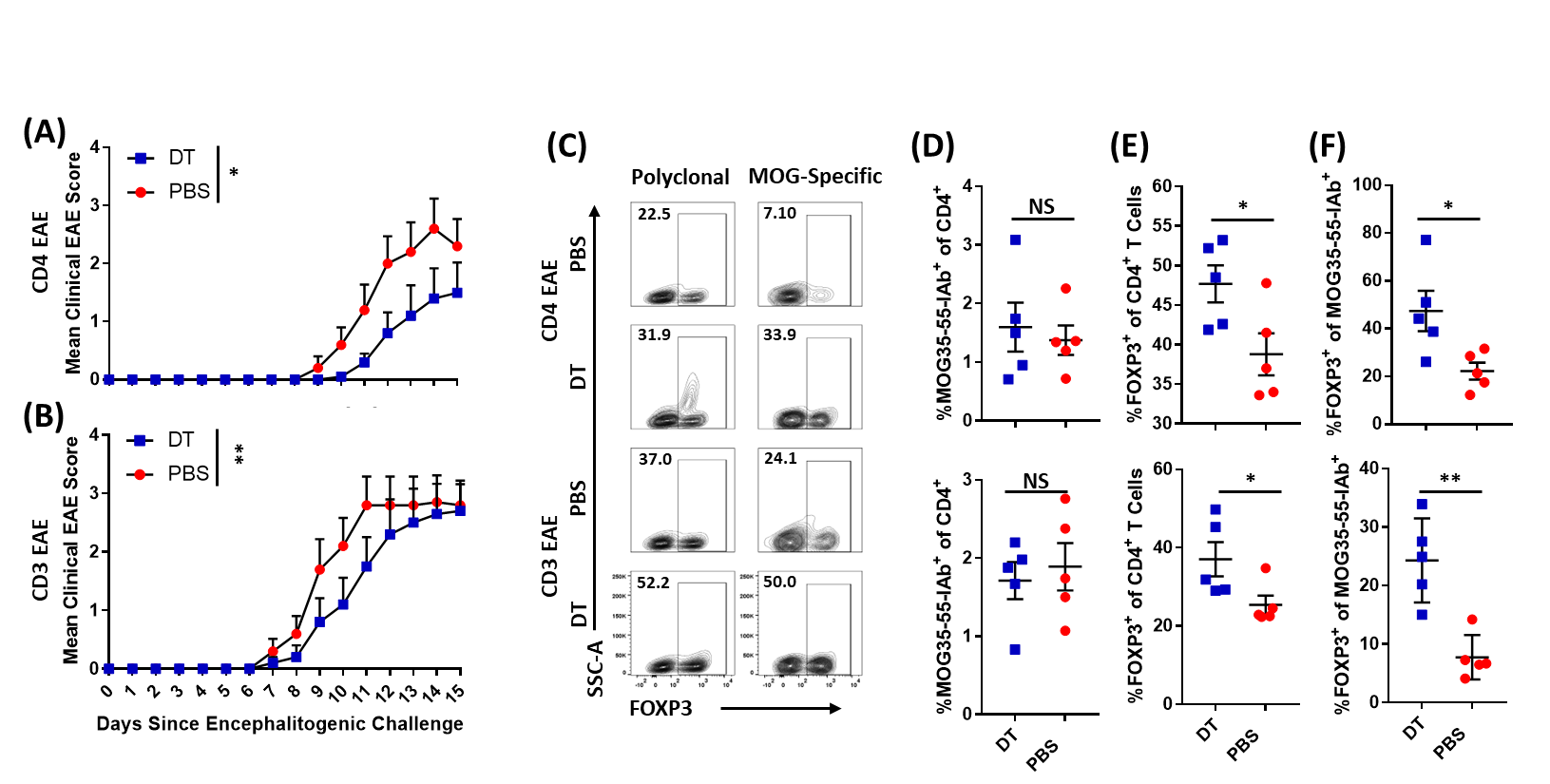


**Supplemental Figure 9: Diphtheria toxin mediated DC1 depletion suppressed the onset of passive CD4^+^ and CD3^+^T cell adoptive transfer EAE in association with increased Treg populations.**

(A-F) XCR1-DTR mice (n=10/group) were IV injected with 0.8^6^ purified CD4^+^ or 2^6^ CD3^+^ encephalitogenic T cells on day 0. Subsequently, mice were injected IP with 120 ng PTx on day 0 and 2 and with 400 ng DT IP every other day from day 0 to 15. Shown (A,B) are the daily mean clinical EAE scores from day 0 through day 15 for DT or PBS treated mice receiving CD4^+^ (top) or CD3^+^ (bottom) encephalitogenic challenge. The CNS (spinal cord and brain) was harvest on day 21 and analyzed via flow cytometry for the presence Tregs. Shown (C) are representational dotplot; first column, T cells (TCRb^+^ CD4^+^) assessed for SSC-A (y-axis) and FOXP3 (x-axis); second column, MOG^35-55^-specpfic CD4^+^ T Cells (CD45.2^+^ TCRb^+^, CD4^+^) assessed for SSC-A (y-axis) and FOXP3 (x-axis). Graphed (D) are the percent MOG35-55-IAb^+^ CD4^+^ T cells and FOXP3^+^ Tregs (E) of the total CD4^+^ T cell pool for mice receiving CD4^+^ (top) or CD3^+^ (bottom) encephalitogenic challenge. Shown (F) are the percent FOXP3^+^ Tregs of the total MOG35-55-scpeicif CD4+ T cells. Statistical significance was analyzed by use of an (F,G and H) unpaired two-tailed t test and (A-D) Wilcoxon matched-pairs test, *p ˂ 0.05, **p ˂ 0.01, ***p ˂ 0.001. Error bars represent SEM.

**Supplemental Figure 10:**


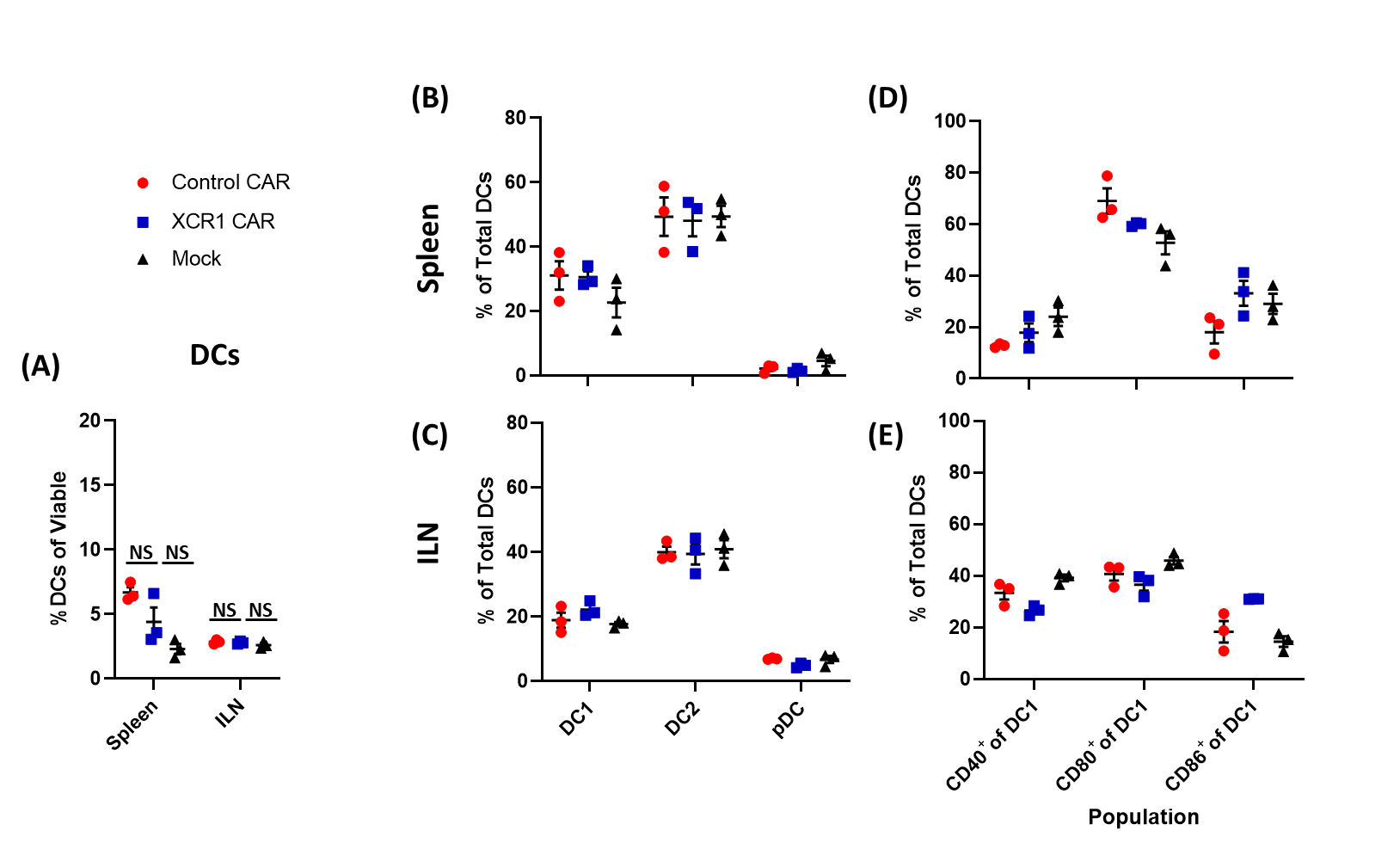


**Supplemental Figure 10: DC subsets following XCR1 CAR-Treg engraftment in RAG2^-/-^ mice.**

(A-E) RAG2^-/-^ mice were iv injected with 3 x 10^6^ XCR1, control or mock transduced CD4^+^ CAR-Tregs and 3 x 10^6^ CD4^+^ T cells (n=3/group). On day 5, spleen and ILN were collected and analyzed via flow cytometric analysis for CAR-T and DC subsets. Graphed (A) are the percent DCs (CD11c^+^ MHCII^+^) of total CD45 for the ILN and spleen. (B, C) The percent DC1 (XCR1^+^ CD11b-), DC2 (CD11b^+^ XCR1^-^) of total DC are shown for the spleen (B) and the ILN (C). Shown (D, E) are the percent CD40^+^, CD80^+^ and CD86^+^ of DC1s.

**Supplemental Table 1:**
